# RNAi screen reveals a role of SPHK2 in dengue virus–mediated apoptosis in hepatic cell lines

**DOI:** 10.1371/journal.pone.0188121

**Published:** 2017-11-16

**Authors:** Atthapan Morchang, Regina Ching Hua Lee, Pa-thai Yenchitsomanus, Gopinathan Pillai Sreekanth, Sansanee Noisakran, Justin Jang Hann Chu, Thawornchai Limjindaporn

**Affiliations:** 1 Division of Molecular Medicine, Department of Research and Development, Faculty of Medicine Siriraj Hospital, Mahidol University, Bangkok, Thailand; 2 Graduate Program in Immunology, Department of Immunology, Faculty of Medicine Siriraj Hospital, Mahidol University, Bangkok, Thailand; 3 Laboratory of Molecular RNA Virology and Antiviral Strategies, Yong Loo Lin School of Medicine, National University Health System, National University of Singapore, Singapore; 4 Medical Biotechnology Unit, National Center for Genetic Engineering and Biotechnology, National Science and Technology Development Agency, Bangkok, Thailand; 5 Department of Anatomy, Faculty of Medicine Siriraj Hospital, Mahidol University, Bangkok, Thailand; Institut Pasteur of Shanghai Chinese Academy of Sciences, CHINA

## Abstract

Hepatic dysfunction is a feature of dengue virus (DENV) infection. Hepatic biopsy specimens obtained from fatal cases of DENV infection show apoptosis, which relates to the pathogenesis of DENV infection. However, how DENV induced liver injury is not fully understood. In this study, we aim to identify the factors that influence cell death by employing an apoptosis-related siRNA library screening. Our results show the effect of 558 gene silencing on caspase 3-mediated apoptosis in DENV-infected Huh7 cells. The majority of genes that contributed to apoptosis were the apoptosis-related kinase enzymes. Tumor necrosis factor superfamily member 12 (*TNFSF12*), and sphingosine kinase 2 (*SPHK2*), were selected as the candidate genes to further validate their influences on DENV-induced apoptosis. Transfection of siRNA targeting *SPHK2* but not *TNFSF12* genes reduced apoptosis determined by Annexin V/PI staining. Knockdown of *SPHK2* did not reduce caspase 8 activity; however, did significantly reduce caspase 9 activity, suggesting its involvement of *SPHK2* in the intrinsic pathway of apoptosis. Treatment of ABC294649, an inhibitor of *SPHK2*, reduced the caspase 3 activity, suggesting the involvement of its kinase activity in apoptosis. Knockdown of *SPHK2* significantly reduced caspase 3 activity not only in DENV-infected Huh7 cells but also in DENV-infected HepG2 cells. Our results were consistent across all of the four serotypes of DENV infection, which supports the pro-apoptotic role of *SPHK2* in DENV-infected liver cells.

## Introduction

Dengue virus (DENV) infection is a mosquito-borne disease, which is characterized by symptoms that range from mild systemic illness to hemorrhagic fever and circulatory shock. Abnormalities in hematologic parameters, including thrombocytopenia and leucopenia, are seen in severe DENV infection [1]. From the site of infection, the viral particles spread to multiple target organs via the circulatory system and lymphatic circulatory system [2].

Hepatic dysfunction is one of the important features of DENV infection. [3]. Liver injury due to hepatocyte apoptosis was observed in severe DENV cases [4–7]. Viral antigens were detected in hepatocytes and Kuppfer cells in patients with hepatomegaly and raising level of serum transaminases [8–12]. BALB/c mouse models of DENV infection [13–15] revealed that high levels of apoptosis were found in livers with high viral load [13, 14, 16]. World Health Organization (WHO) guideline suggested organ injury as one of the criteria for determining severity of DENV disease [17]. Viral components, including DENV membrane (DENV M) and capsid (DENV C), were found to contribute to apoptosis [18–20]. DENV induces hepatocyte apoptosis via caspase 8 and 9 suggests the involvement of both intrinsic and extrinsic pathways of apoptosis. The extrinsic pathway involves extracellular death ligands-receptors signaling such as tumor necrosis factor α (TNF-α) signaling whereas the intrinsic pathway activates the mitochondrial membrane permeabilization (MMP) event, which is triggered by intracellular stress, such as endoplasmic reticulum stress and oxidative stress [21]. Both intrinsic and extrinsic pathways contribute to caspase 3 activation both *in vitro* cultures [22, 23] and in animal models [13, 14].

Delivery of gene-specific small interfering RNA (siRNA) is a transient gene silencing tool that is widely used to investigate the biological function of a gene of interest [24]. The combination of siRNAs and a high-throughput screening platform can help to identify how multiple genes contribute to a specific molecular signaling mechanism [25]. Genome-scale knockdown experiments in flaviviral infections have been conducted by several research groups [26–28]. Two of these groups characterized a number of host factors that are mutually required for mosquito-borne flavivirus infections, including DENV, West Nile virus (WNV), and yellow fever virus (YFV) [26, 28], while the other group demonstrated the important host factors required for DENV to infect insect cells [27]. Interestingly, a pathway-focused siRNA library screening experiment explained the role of human trafficking genes in DENV entry to the host cells [29]. However, apoptosis siRNA library screening to identify genes required for DENV-induced apoptosis has never been investigated.

In this study, we employed an apoptosis pathway-focusing siRNA library, which contains a smart pool of 558 siRNAs targeting apoptotic genes to identify the genes, which were involved in apoptosis based on the level of caspase 3 activity in DENV-infected Huh7 cells. Our results show that *SPHK2* contributes to DENV-mediated apoptosis in hepatic cells.

## Materials and methods

### Cell culture and preparation of DENV

Huh7 cells were obtained from the Japanese Collection of Research Bioresources Cell Bank (JCRB0403). HepG2 cells and A549 cells were obtained from American Type Culture Collection (ATCC, Manassas, VA, USA). All cells were maintained in Dulbecco’s Modified Eagle Medium (DMEM) (Gibco; Thermo Fisher Scientific, Inc., Waltham, MA, USA) supplemented with 10% heat inactivated fetal bovine serum (FBS) (Gibco; Thermo Fisher Scientific, Inc., Waltham, MA, USA) and 100 U/ml of penicillin and streptomycin in 37°C, 5% CO_2_ and humidified incubator. DENV serotype 1 strain Hawaii, serotype 2 strain 16881, serotype 3 strain H87, and serotype 4 strain H241 were propagated in C6/36 mosquito cell lines. Briefly, confluent monolayers of C6/36 cells were separately infected with the four serotypes of DENV at a multiplicity of infection (MOI) of 10. Six days after infection, the supernatants were collected by centrifugation at 5,000 rpm for 10 minutes. Virus titer was quantified according to standard plaque forming unit (PFU) assay using BHK-21 cells. Supernatant containing virus was aliquoted in microcentrifuge tubes and frozen at -80°C until use.

### Assay for cell viability and caspase 3 activity

Huh7 cells were seeded at 1.5 x 10^4^ cells in a 96-well white plate with a clear bottom for one day before infection. Cells were inoculated with DENV at MOI 1, 5, and 10 for 2 hours. Cells were then replenished with 100 μl of DMEM maintenance media containing 2% FBS and 100 U/ml of penicillin and streptomycin. Cells were cultured for 24, 48, 72, and 96 hours post infection (hpi). Morphological cell death was observed using phase-contrast light microscopy. Cell viability and caspase 3 activity were determined using ApoLive-Glo^™^ Multiplex Assay (Promega Corporation, Madison, WI, USA). This assay includes a substrate for both live-cell protease and caspase 3 protease, which are used to measure cell viability and apoptosis as a proportional fluorescent and luminescent signal, respectively. Briefly, 10 μl of viability reagent was added to cells and incubated at 37°C for 1 hour. Fluorescent signal was measured at 400_ex_-500_em_ nanometer using an Infinite 200 PRO microplate reader (Tecan Group Ltd., Männedorf, Switzerland). Thereafter, 100 μl of caspase 3 activity reagent was added and incubated for 1 hour at room temperature. Luminescent signals were analyzed using a GloMax^®^-96 Microplate Luminometer (Promega Corporation, Madison, WI, USA). Data were presented in relative fluorescent units (RFUs) and relative light units (RLUs) or percentage compared to control group.

### Reverse transfection of siRNAs in Huh7 cells

Transfection was performed using DharmaFECT 4 transfection reagent (GE Dharmacon, Lafayette, CO, USA) in a 96-well white plate with a clear bottom. A smart pool of non-targeting control (NTC) siRNA (D-001206-13; GE Dharmacon) and caspase 3 siRNA (L-004307-00; GE Dharmacon) were used as negative and positive control, respectively. siRNA was diluted in DharmaFECT cell culture reagent (GE Dharmacon) and used at a final concentration of 50 nM. Transfection reagent and siRNA were mixed and incubated at 25°C for 30 minutes to form siRNA-liposome complex. Huh7 cells at 1.5 x 10^4^ cells per well were allowed to plate onto the transfection mixture and were then incubated for 24 hours. The media were then aspirated out and the transfected cells were infected with supernatant containing DENV at MOI 10 and incubated for 48 hours. Cell viability and caspase 3 activity were measured as previously described.

### siRNA library

Human ON-TARGETplus^®^ siRNA Library—Apoptosis—SMART pool (Catalogue #G-103900-E2-01) was purchased from GE Dharmacon (Lafayette, CO, USA). The library contains a smart pool of siRNAs that includes four siRNA duplexes design to target each of the 558 apoptosis-related genes. This allows specific knockdown with high efficiency, as well as prevention of gene compensation of a specific isotype. Deconvolution of this smart pool in the following experiment demonstrated the specific knockdown effect mediated by these siRNAs.

### siRNA library screening

Lyophilized siRNAs in the library were resuspended in DEPC-treated water to make a stock concentration of 100 μM. Stock siRNA was diluted to working concentration of 5 μM freshly before experiments. Transfection was performed in a 384-well white plate with a clear bottom and the final concentration of each siRNA is 50 nM. For each well, 0.04 μl of transfection reagent and 0.5 μl of 5 μM working siRNA were separately diluted in 2 μl of DharmaFECT cell culture transfection media. siNTC was used as the negative control. After separately incubation at 25°C for 5 minutes, the reagents were combined to form siRNA-liposome complex at 25°C for 30 minutes. At optimized conditions, 2 x 10^3^ Huh7 cells in 46 μl of growth media were added directly to the transfection mixture and incubated for 24 hours to ensure knockdown efficiency. The media were then aspirated out and the transfected cells were infected with 50 μl of supernatant containing DENV at MOI 10 and further incubated for 48 hours. Cell viability and caspase 3 activity were measured, as previously described. All liquid handling steps were performed using Multidrop^™^ Combi Reagent Dispenser (Thermo Fisher Scientific, Inc., Waltham, MA, USA) and Embla Microplate Washer (Molecular Devices, LLC, Sunnyvale, CA, USA).

### Real-time RT-PCR

Huh7 cells were transfected with either 50 nM of siNTC or 50 nM of si*TNFSF12* (5’- GCUCCUCCCUUGAGAAUUC-3’, J-010629-08; GE Dharmacon, Lafayette, CO, USA) and si*SPHK2* (5’-GAGACGGGCUGCUCCAUGA-3’, J-004831-10; GE Dharmacon, Lafayette, CO, USA). Total RNA was isolated from transfected cells using High Pure RNA Isolation Kit (Roche Applied Science, Penzberg, Germany). RNAs concentration and purity were measured using a Nano Drop ND-1000 Spectrophotometer (Thermo Fisher Scientific, Inc., Waltham, MA, USA). Equivalent concentrations of RNAs were reverse transcribed to cDNA using SuperScript^®^ III Reverse Transcription System (Invitrogen, Carlsbad, CA, USA). cDNAs were then mixed with LightCycler^®^ 480 SYBR Green I Master Mix (Roche Diagnostics, Basel, Switzerland) and individual primer sets for amplification. The following primers were used in this study: *TNFSF12-F*: 5’—CCT CGC AGA AGT GCA CCT AAA—3’; *TNFSF12-R*: 5’—TCA GGT AGA CAG CCT TCC CC—3’; *SPHK2-F*: 5’- CTG ACT AGC CGG GCG ATA AC—3’; *SPHK2-R*: 5’- CCT GAC CTT CAG CTC TCC AAC—3’; *ACTB-F*: 5’- AGA AAA TCT GGC ACC ACA CC—3’; and, *ACTB-R*: 5’- CTC CTT AAT GTC ACG CAC GA—3’. PCR amplification was performed on a LightCycler^®^ 480 Real-Time PCR System (Roche Applied Science, Penzberg, Germany) with a program profile of pre-incubation at 95°C for 10 minutes, followed by 40 cycles of denaturation at 95°C for 10 seconds, annealing at 60°C for 10 seconds, and extension at 72°C for 20 seconds. Gene fold change was calculated according to 2^-ΔΔCt^ values compared between test and control.

### Apoptosis assay using Annexin V/propidium iodide staining

Apoptosis assay was conducted using BD Pharmingen^™^ Annexin V-FITC Apoptosis Detection Kit I (Bectin, Dickinson and Company, Franklin Lakes, NJ, USA). Briefly, siRNA transfected and DENV-infected Huh7 cells were harvested and washed with cold 1X BD Pharmingen^™^ Annexin V Binding Buffer. Cells were then resuspended in 100 μl of 1X Annexin V Binding Buffer and incubated with 5 μl of Annexin V-FITC on ice in the dark for 15 minutes. Thereafter, 2.5 μl of propidium iodide (PI) was added and the final volume of reaction was adjusted to 300 μl. Stained cells were immediately subject to analysis using FACSORT^™^ (Bectin, Dickinson and Company). Green channel (FL-1) and red channel (FL-2) were used to detect Annexin V-FITC and PI, respectively. Annexin V-positive/PI-negative cells were considered to be apoptotic cells and were analyzed as a percentage of the entire cell population.

### Assays for caspase 8 and caspase 9 activities

The cells were subjected to measure the activity of caspase 8 and caspase 9 using a specific-substrates, luminescent-based commercial kit, Caspase-Glo^®^ 8 Assay and Caspase-Glo^®^ 9 Assay Systems from Promega. Briefly, the cells in 100 μl of culture medium were combined with 100 μl of Caspase-Glo^®^ 8 or Caspase-Glo^®^ 9 working reagents. The reagents provide lysis buffer, luminescent-conjugated substrates of caspase8 or caspase 9 and MG132. MG132 was added to lower the background signal. The reaction mix was incubated for 30 minutes at room temperature and total luminescent light was measured using Synergy H1 Multi-Mode Reader (BIOTEK, Winooski, VT, USA)

### Treatment of ABC294640, a SPHK2 inhibitor

ABC294640 was purchased from Echelon Biosciences, Inc. (Salt Lake City, UT, USA) and dissolved in DMSO to create a 10 mM concentration of inhibitor stock, which was stored at -20°C in a dark condition until use. The working solution was freshly prepared in 2.5, 5, and 10 μM concentrations before treatment. Growth media and 0.01% DMSO were used as parental and vehicle controls, respectively. For cytotoxicity testing, Huh7 cells were seeded into 96-well culture plates, treated with ABC294640 at different concentrations, and incubated for 48 hours. Cell viability was measured using PrestoBlue Assay (Invitrogen Carlsbad, CA, USA). For determination of caspase 3 activity and sub G1 cells population, Huh7 cells were pre-treated with 2.5, 5, and 10 μM concentrations of ABC294640 for 2 hours. Cells were then washed with PBS and inoculated with DENV at MOI 10 for 2 hours. After washing, the cells were cultured in the presence or absence of ABC294640 for 48 hours. Caspase 3 activity was then measured as previously described. Sub G1 cells population was detected by fixing the cells in ice-cold 70% ethanol overnight, followed by treatment of 2 mg/ml of RNAse in 1% Triton X-100 for 5 minutes at room temperature. The cells were stained with 10 μg/ml of PI for 30 minutes at room temperature in the dark. The stained cells were subject to analysis using a flow cytometer. Red channel (FL-2) was used to detect DNA content, which was stained by PI.

### Immunofluorescent assay (IFA)

Cells were seeded on a glass cover slip for 24 hours before experiment. HepG2 cells were infected with DENV at the MOI of 5. Huh7 cells and A549 cells were infected with DENV at the MOI of 1. After 24 hours post infection, cells were fixed by incubation with 4% paraformaldehyde at room temperature for 20 minutes. Cells were washed and permeabilized by incubation with 0.2% Triton-X (Sigma-Aldrich Corporation, St. Louis, MO, USA) at room temperature for 10 minutes. Thereafter, cells were incubated with both mouse anti-DENV E monoclonal antibody (Clone 4G2) and rabbit anti-SPHK2 (ab37977, Abcam) at a dilution of 1:50 at 37°C for 60 minutes. Cells were washed and incubated with secondary antibodies containing goat anti-mouse IgG-Cy3 (A10521, Molecular Probes) and donkey anti-rabbit IgG-Alexa 488 (A21206, Molecular Probes) at a dilution of 1:500 at 37°C in the dark for 1 hour. Hoechst 33342 solution (H3570, Molecular Probes) was added at a dilution of 1:1000 for nuclear detection. After the washing step, cover slips were mounted onto a glass slide using 50% glycerol, sealed, and subject to fluorescent image capture using confocal microscopy (LSM 510 Meta; Carl Zeiss Microscopy GmbH, Jena, Germany).

### Statistical analysis

For the siRNA screening experiment, each siRNA reverse transfection was performed in triplicate wells and data were shown as percentage of caspase 3 activity compared to the siNTC control. The rest of the experiments were performed at least three independent experiments. Data were analyzed using GraphPad Prism Software version 5 (GraphPad Software, Inc., San Diego, CA, USA) and presented as mean ± standard deviation (SD). Statistical analysis was performed using Student’s unpaired *t*-test. A *p*-value less than 0.05 was considered to be statistically significant.

## Results

### DENV infection induces Huh7 cells apoptosis in a time- and dose-dependent manner

**Fig 1:**
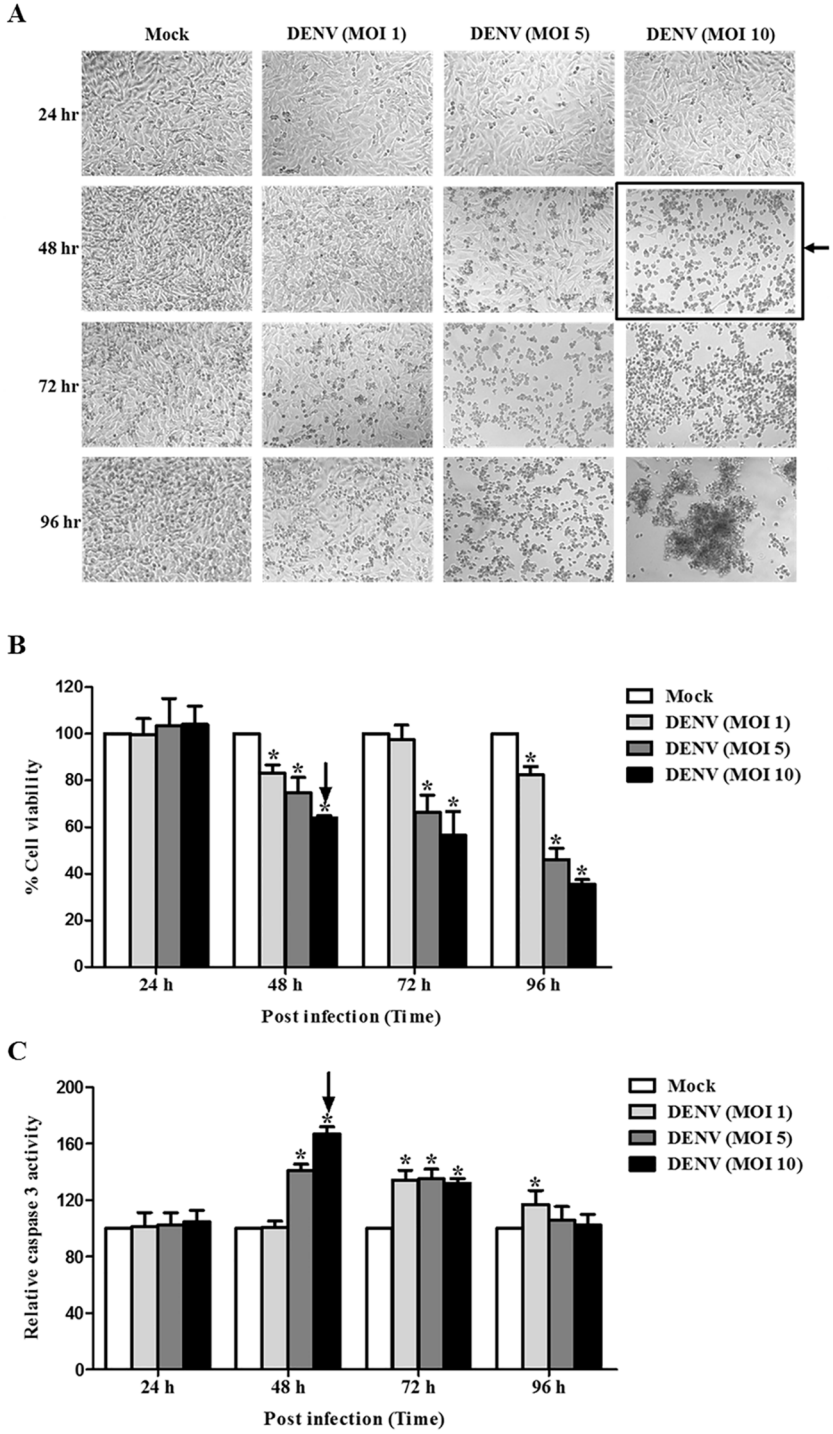
DENV-infected Huh7 cells undergo apoptosis in a time- and dose-dependent manner. Huh7 cells were infected with DENV at MOIs of 1, 5, and 10 and incubated at different time points of 24, 48, 72, and 96 hpi. Cell morphology was monitored using phase contrast light microscopy. Cell viability and caspase 3 activity were measured using multiplex detection kit: (A) Cell morphology; (B) Cell viability; and (C) Caspase 3 activity are shown. For (B) and (C), the results are expressed as percentage to that of mock control that obtained from the average of three independent experiments ± SD. The asterisks indicate statistically significant differences between groups (p < 0.05) (Student’s *t* test). The arrow indicated the condition chosen for screening assay.

To determine the apoptosis inducing condition, Huh7 cells were infected with DENV MOI 1, 5, and 10 and were incubated for 24, 48, 72, and 96 hpi. Cell morphology, cell viability, and caspase 3 activity were determined to characterize DENV-induced cell death. No change in cell morphology was observed at 24 hpi (Fig 1A), whereas shrinking, rounding, and floating of cells were visualized at 48 hpi for MOI 5 and 10. Cell death became more obvious at 72 and 96 hpi, with commensurate increases being observed at higher MOI (Fig 1A). Decreased cell viability was consistently observed at all-time points from 48 hpi to 96 hpi (Fig 1B). As expected, increased caspase 3 activity became observable at 48 hpi for MOI 5 and 10, but it declined at 72 and 96 hpi (Fig 1C). These results suggest that DENV infection of Huh7 cells induced apoptosis in both a time-dependent and MOI-dependent manner. The apoptosis inducing condition for the screening was determined to be 48 hpi at DENV MOI 10, since the maximum increase in caspase 3 activity was observed at those parameters.

### Optimization of siRNA screening platform

**Fig 2:**
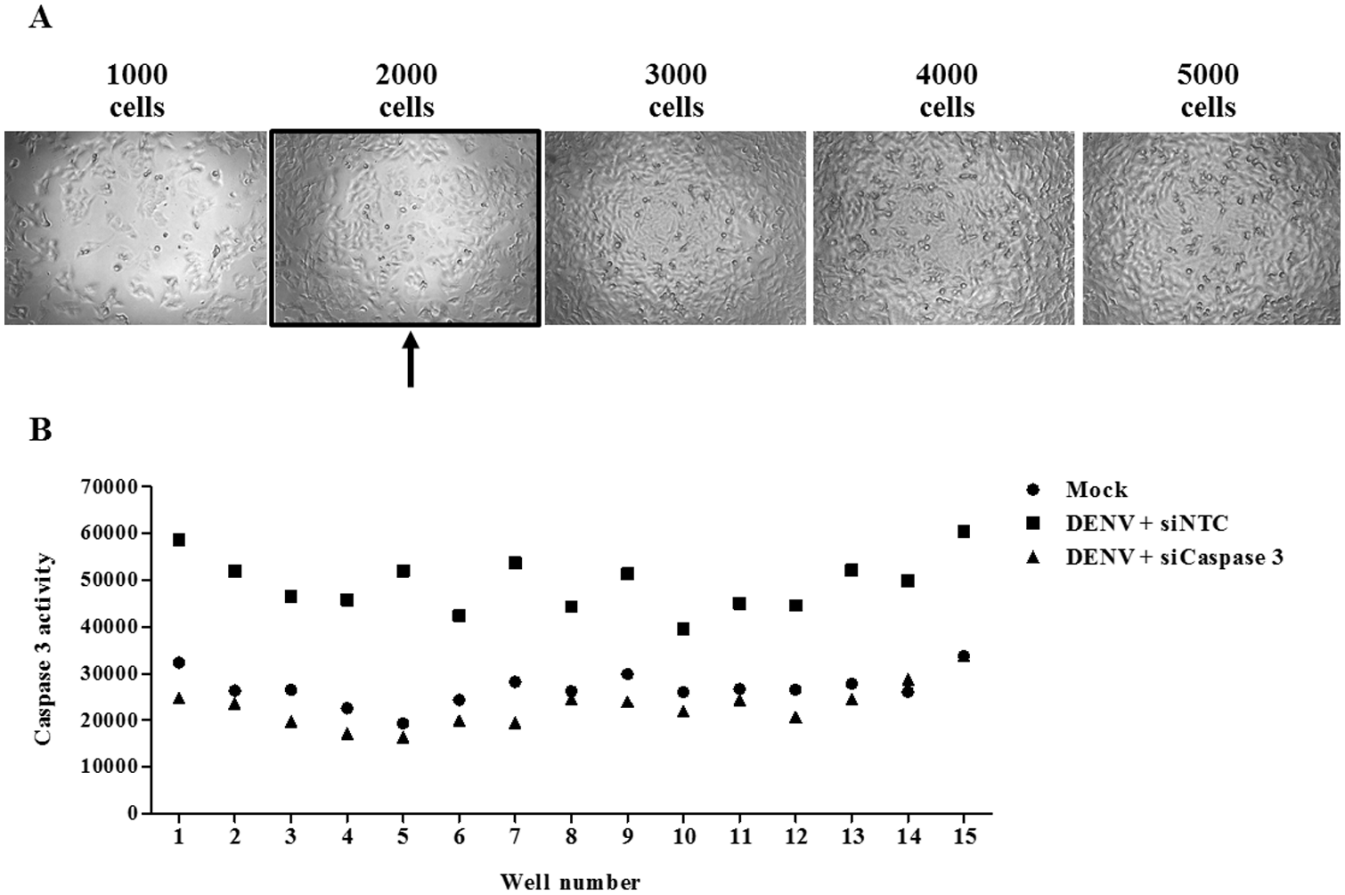
Optimized siRNA screening condition. (A) Huh7 cells were seeded into a 384-well plate with 1000, 2000, 3000, 4000, and 5000 cells per well. Cell morphology and confluency were observed and an image was captured 72 hours later using a phase contrast microscopy. The arrow indicates the optimized seeding condition for siRNA screening assay. (B) Huh7 cells were reverse transfected with a siNTC or si*Caspase* 3 for 24 hours before infected with DENV at MOI of 10 for 48 hpi. Conditions were replicated in 15 wells within the same plate. Caspase 3 activity was measured and plotted across the 15 wells.

Optimization of a suitable siRNA screening platform according to the parameters of 48 hpi at DENV MOI of 10 was conducted. Since siRNA screening was miniaturized into a 384-well plate format, the seeding condition had to be optimized first. Huh7 cells were seeded at a density of 1000, 2000, 3000, 4000, and 5000 cells and then cultured for 72 hours. Cell morphology and confluency were then studied under a light microscope. Our results show progressive cell growth and spontaneous cell death in wells seeded with more than 2000 cells (Fig 2A). However, the seeding condition of 2000 cells per well showed an appropriate density and monolayer culture. As a result, 2000 cells per well was selected as the seeding condition for the experiments in this study.

To evaluate the accuracy and reliability of the screening, we undertook knockdown experiments with siRNA specific to caspase 3 as a positive control. Cells were reverse transfected with a non-targeted control siRNA (siNTC) or caspase 3 siRNA (si*Caspase* 3) for 24 hours and then infected with DENV MOI 10 for 48 hours. The results from 15 repeated caspase 3 knockdown wells precisely showed the reduction in caspase 3 activity upon DENV infection (Fig 2B).

### siRNA library screening identifies genes, which affect caspase 3 activity in DENV-infected Huh7 cells

The SMART pool apoptosis human siRNA library comprises siRNAs that target apoptosis-related genes for the comprehensive analysis of apoptotic signaling. The siRNA targets include both cytoplasmic and membrane-bound proteins associated with programmed cell death. A SMART pool of 4 different siRNA duplexes designs for an individual gene to enhance knockdown efficiency. Complete list of the alteration of caspase 3 activity in DENV-infected Huh7 cells affected by siRNA library transfection can be found at S1 Table. Our siRNA library screening verified various genes related to apoptotic signals. Based on the highest ability to reduce caspase 3 activity in DENV-infected Huh7 cells, 20 siRNA targeted genes were reported in Table 1. Tumor necrosis factor superfamily, member 12 (*TNFSF12*), which is known as TNF-related weak inducer of apoptosis (*TWEAK*), show the strongest reduction of caspase 3 activity upon silencing. Nine of the 20 genes selected by having the most reduction in caspase 3 activities were categorized as kinase enzymes, including *STK17A*, *STK17B*, *CARD11*, *CARD14*, *NME5*, *NME3*, *RIPK2*, and *SPHK2*. siRNAs targeted genes encoding caspase 3 and cytochrome C, which are the known regulators of apoptosis, were also shown to reduce caspase 3 activity.

**Table 1:** List of top 20 genes identified from siRNA library screening.

	Gene symbol	Gene description	Percentage of caspase 3 activity (compare to siNTC)	S.D.	p-value
1	*TNFSF12*	Tumor necrosis factor ligand superfamily member 12	44.25%	6.95	0.0002
2	*RIPK2*	Receptor-interacting serine/threonine protein kinase 2	45.02%	13.36	0.0022
3	*STK17A*	Serine/threonine kinase 17a	47.46%	7.58	0.0003
4	*CARD14*	Caspase recruitment domain family member 14	48.12%	6.05	0.0001
5	*PDCD2*	Programmed cell death-2	48.51%	4.56	< 0.0001
6	*HDAC3*	Histone deacetylase 3	48.82%	7.15	0.0003
7	*PSEN2*	Presenilin 2	48.99%	6.86	0.0002
8	*PIM1*	Serine/threonine-protein kinase Pim-1	51.28%	17.36	0.0374
9	*SST*	Somatostatin	52.20%	14.23	0.0047
10	*RAD21*	RAD21 homolog (*S*. *pombe*)	53.45%	11.91	0.0027
11	*SPHK2*	Sphingosine kinase 2	54.68%	16.96	0.0107
12	*RASA1*	RAS P21 protein activator (GTPase activating protein) 1	54.71%	1.79	< 0.0001
13	*NME3*	NME/NM23 nucleoside diphosphate kinase 3	54.96%	8.82	0.0010
14	*CASP3*	Caspase 3, apoptosis-related cysteine peptidase	55.20%	8.89	0.0009
15	*CYCS*	Cytochrome C	55.76%	7.28	0.0005
16	*NME5*	NME/NM23 nucleoside diphosphate kinase 5	56.02%	8.31	0.0008
17	*CARD11*	Caspase recruitment domain family member 11	56.23%	2.63	< 0.0001
18	*STK17B*	Serine/threonine kinase 17b	57.00%	9.32	0.0014
19	*SIVA1*	SIVA1, Apoptosis-Inducing Factor	57.09%	8.50	0.0010
20	*CTNNAL1*	Catenin alpha-like protein	57.13%	8.03	0.0008

### *SPHK2* knockdown reduces DENV-induced apoptosis in Huh7 cells

**Fig 3:**
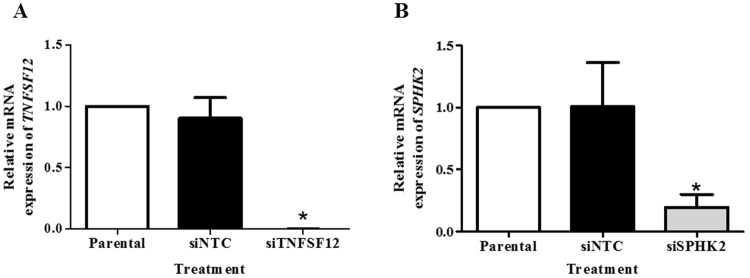
Knockdown efficiency of *TNFSF12* and *SPHK* in DENV-infected Huh7 cells. Huh7 cells were reverse transfected with siRNA targeted *TNFSF12* or *SPHK2* genes or the non-targeting control siRNA for 24 hours before infected with DENV for 48 hours. The mRNA expression was then analyzed using real-time RT-PCR. The mRNA expression of *TNFSF12* (A) and *SPHK2* (B) represented as fold times compared to parental control. The results are expressed as the average of three independent experiments ± SD. The asterisks indicate statistically significant differences between groups (p < 0.05) (Student’s *t* test).

*TNFSF12* and *SPHK2* were selected for further analysis as the contribution of these genes in DENV-mediated apoptosis has never been investigated in DENV infection. To demonstrate the knockdown efficiency, specific mRNA expression in si*TNFSF12*- and si*SPHK2*-transfected Huh7 cells were determined using real-time RT-PCR. Both mRNA expression of *TNFSF12* and *SPHK2* were more than 50% silenced in Huh7 cells after transfection with their specific siRNAs, as compared to rates observed after reverse transfection with siNTC (Fig 3A and 3B) suggesting the efficiency of the knockdown assay.

**Fig 4:**
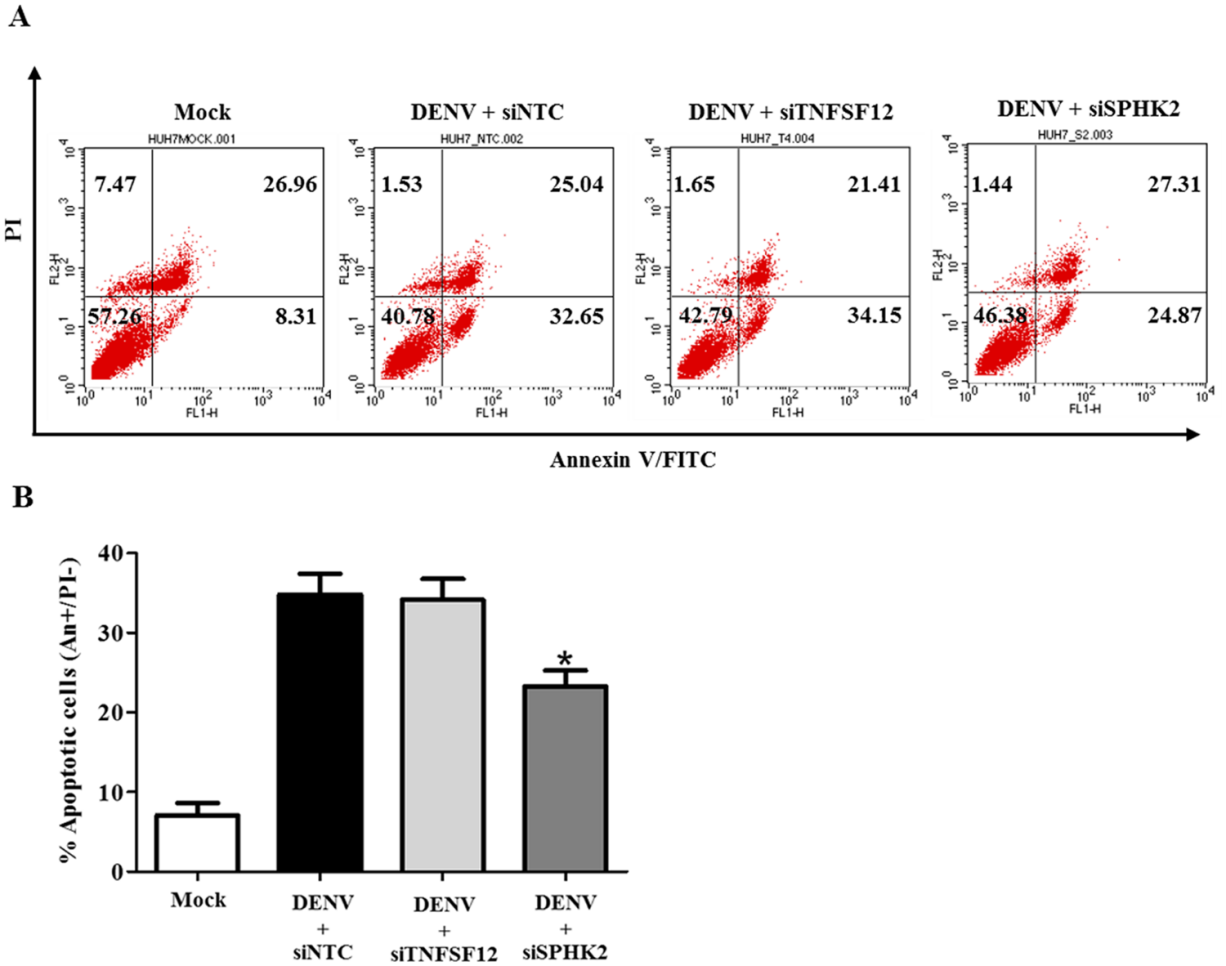
Knockdown of *SPHK2* but not knockdown of *TNFSF12* reduces apoptosis in DENV-infected Huh7 cells. Huh7 cells were reverse transfected with siRNA directed against *TNFSF12* or *SPHK2* genes for 24 hours before being infected with DENV for 48 hours. Apoptotic cells were determined by Annexin V/PI staining and flow cytometry analysis. (A) Dot plot indicates cell death; and, (B) Apoptotic cells (Annexin V+/PI-) were plotted and compared with percentage of cell population. The results are expressed as the average of three independent experiments ± SD. The asterisks indicate statistically significant differences between groups (p < 0.05) (Student’s *t* test).

Annexin V/PI staining was performed to confirm the role of *TNFSF12* and *SPHK2* in the apoptosis of DENV-infected Huh7 cells. Our results show that DENV infection in Huh7 cells induced apoptosis, while reverse transfection with siSPHK2 significantly reduced the rate of apoptotic cell death (Annexin V+/PI-) from 32.66% to 24.87% compared to that of the siNTC transfection control (Fig 4A). However, silencing of *SPHK2* reduced the proportion of primary apoptotic cells, not that of secondary necrotic cell population. In contrast to result of *SPHK2*, reverse transfection with siTNFSF12 was not able to reduce the rate of apoptosis (32.66% to 34.15%) (Fig 4A). Statistical analysis of data from three independent experiments is shown in Fig 4B.

### *SPHK2* knockdown in DENV-infected Huh7 cells modulates the intrinsic pathway of apoptosis

**Fig 5:**
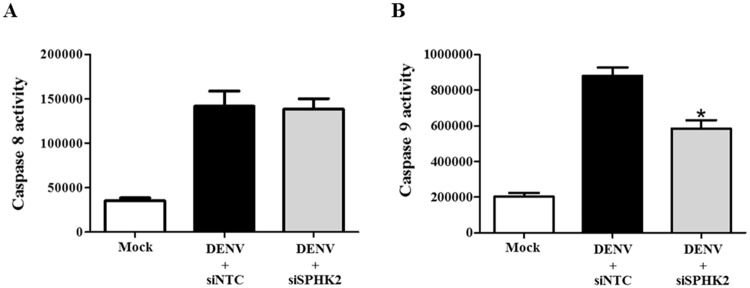
*SPHK2* knockdown modulates apoptosis in DENV-infected Huh7 cells via the intrinsic pathway. Huh7 cells were reverse transfected with si*SPHK2* or siNTC for 24 hours and then infected with DENV MOI 10 for 48 hours. Caspase 8 (A) and caspase 9 (B) activities were determined and measured and the values are represented in relative luminescent unit (RLU). The results are expressed as the average of three independent experiments ± SD. The asterisks indicate statistically significant differences between groups (p < 0.05) (Student’s *t* test).

To gain insight into how *SPHK2* modulates apoptosis in DENV-infected Huh7 cells, the activities of caspase 8 and caspase 9, which represented extrinsic and intrinsic pathways of apoptosis were investigated. Elevated caspase 8 activity (Fig 5A) and caspase 9 activity (Fig 5B) was observed in siNTC-transfected DENV-infected Huh7 cells. Knockdown of *SPHK2* in DENV-infected Huh7 cells significantly reduced caspase 9 activity without affecting caspase 8 activity (Fig 5B and 5A). These results explain the contributory role of *SPHK2* in the intrinsic pathway of apoptosis in DENV-infected Huh7 cells.

### ABC294640, a selective inhibitor of SPHK2, reduces DENV-induced apoptosis in Huh7 cells

**Fig 6:**
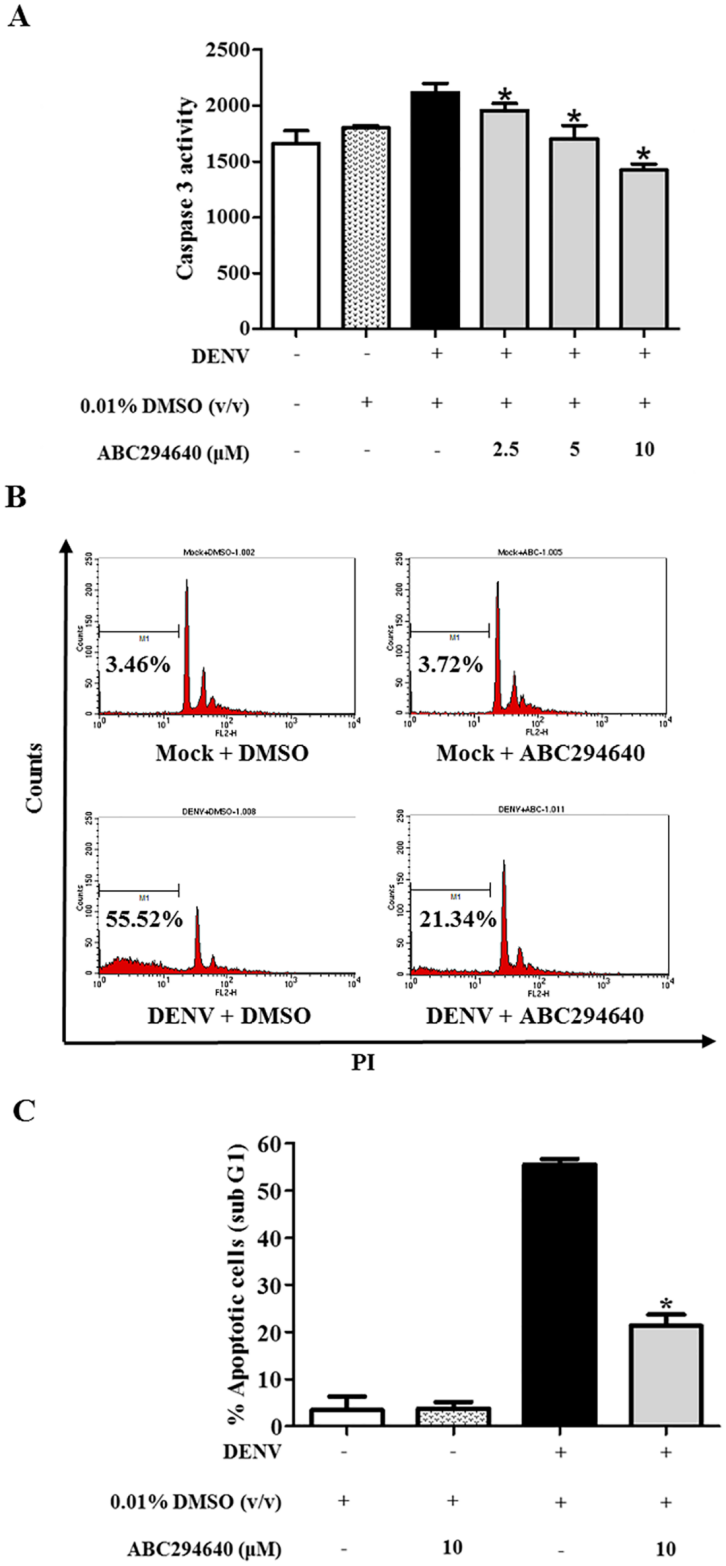
ABC294640 treatment reduces apoptosis in DENV-infected Huh7 cells. Huh7 cells were pre-treated with 0.01% v/v DMSO or 2.5, 5, and 10 μM concentrations of ABC294640 for 2 hours. The treated cells were infected with DENV at MOI 10 and were cultured in the presence of corresponding concentrations for 48 hours. Caspase 3 activity was measured and is represented in relative luminescent unit (RLU) (A). Huh7 cells were pre- and post-infection treated with 0.01% v/v DMSO or 10 μM concentration of ABC294640. Sub G1 cells population was detected by PI staining and flow cytometry at 48 hpi. (B) Histogram plot of sub G1 cells and (C) bar graph represents the average percentage of three independent experiment ± SD. The asterisks indicate statistically significant differences between groups (p < 0.05) (Student’s *t* test).

To determine whether the kinase activity of SPHK2 has any influence on the DENV-induced apoptosis of Huh7 cells, studies with ABC294640, a selective inhibitor of SPHK2, were performed. Cytotoxicity of ABC294640 was initially studied to determine the effective dose. Both ABC294640 and its solvent control (0.01% DMSO) were found to be non-toxic to Huh7 cells at the tested concentrations of 2.5, 5, and 10 μM (S1 Fig). Pre- and post-treatment with ABC294640 showed significant reduction in caspase 3 activity of DENV-infected Huh7 cells in a dose-dependent manner (Fig 6A). Measurement of sub G1 cells, a representative of the DNA fragmented apoptotic cells, consistently showed the significant reduction of apoptosis from 55.52% in untreated cell to 21.34% in ABC294640-treated DENV-infected Huh7 cells (Fig 6B and 6C). These results suggest that the kinase activity of SPHK2 affects the DENV-induced apoptosis of Huh7 cells.

### Knockdown of *SPHK2* does not affect virus production and viral protein expression

**Fig 7:**
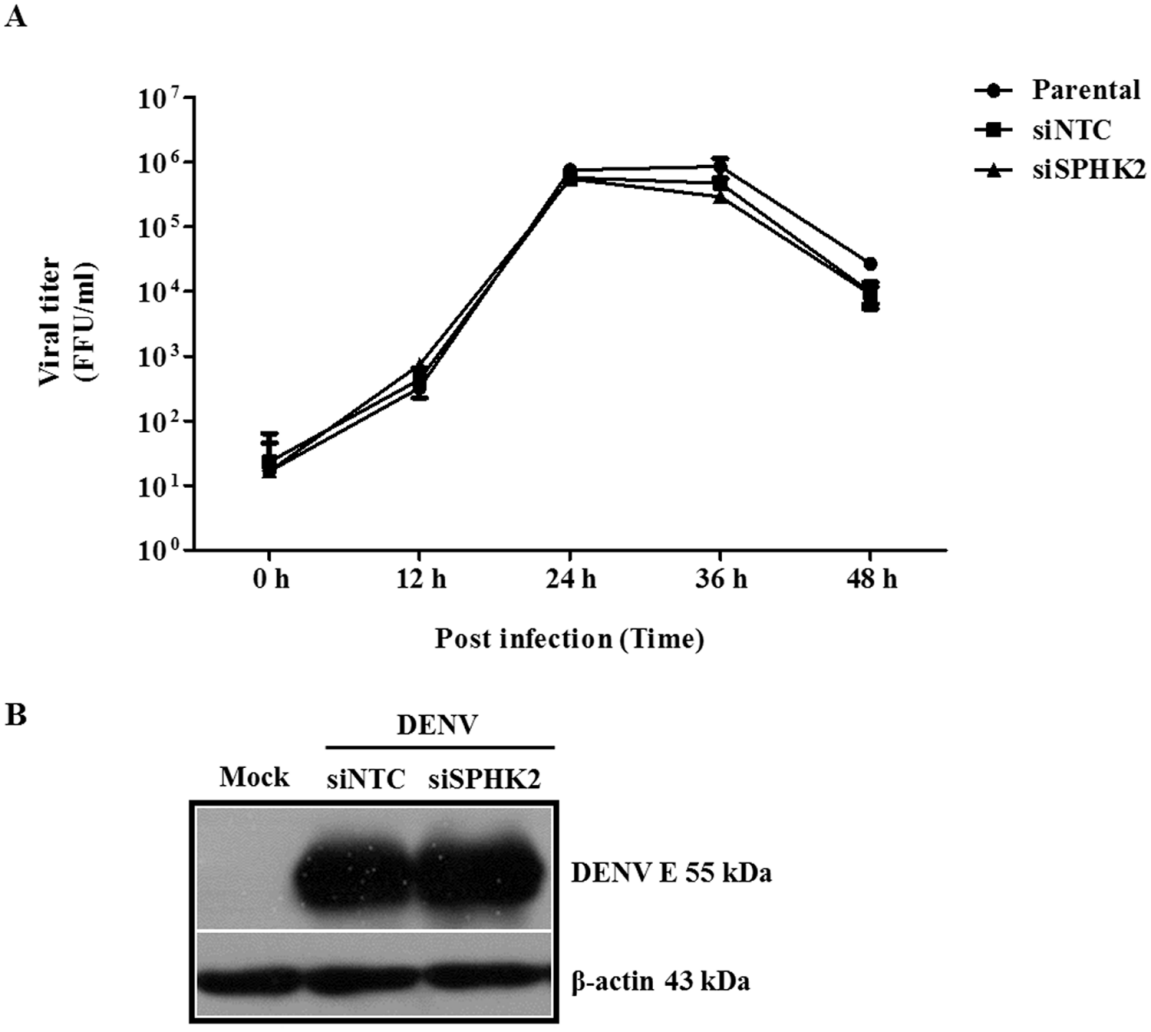
Knockdown of *SPHK2* does not interfere with virus production and viral protein synthesis. Huh7 cells were reverse transfected with siRNA targeted *SPHK2* gene for 24 hours before being infected with DENV for 0, 12, 24, 36 and 48 hours. (A) Virus production in supernatant was quantified by FFU assay. (B) DENV E protein expression was determined at 48 hpi using western blot analysis. The results are expressed as the average of three independent experiments ± SD. The asterisks indicate statistically significant differences between groups (p < 0.05) (Student’s *t* test).

To exclude the possibility that knockdown of *SPHK2* impeded the virus infection thus indirectly affected apoptosis, the kinetic of virion production as well as the level of viral protein synthesis were determined in *SPHK2* knockdown cells. The results show that no significant difference in kinetic of virus production was observed between siNTC and si*SPHK2*-transfected cells over 0–48 hpi (Fig 7A). In consistency, DENV envelope (DENV E) expression was not affected by the transfection of siSPHK2 (Fig 7B). These results indicate that knockdown of *SPHK2* gene does not perturb the activity of DENV infection of Huh7 cells.

### *SPHK2* knockdown reduces apoptosis in DENV-infected hepatic cell lines

**Fig 8:**
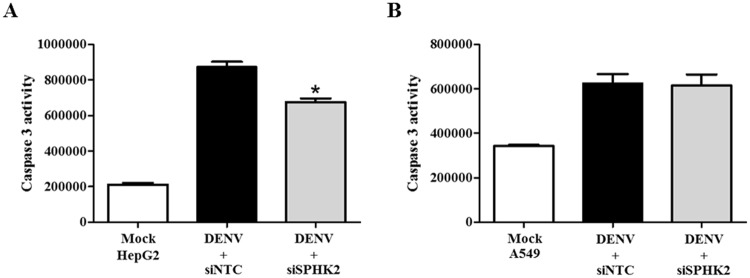
*SPHK2* knockdown reduces apoptosis in HepG2 cells but not in A549 cells. HepG2 cells and A549 cells were reverse transfected with si*SPHK2* or siNTC for 24 hours and then infected with DENV MOI 10 for 48 hours. Caspase 3 activity was measured and represented for (A) HepG2 cell and (B) A549 cells. The results are represented in relative luminescent unit (RLU). The results are expressed as the average of three independent experiments ± SD. The asterisks indicate statistically significant differences between groups (p < 0.05) (Student’s *t* test).

**Fig 9:**
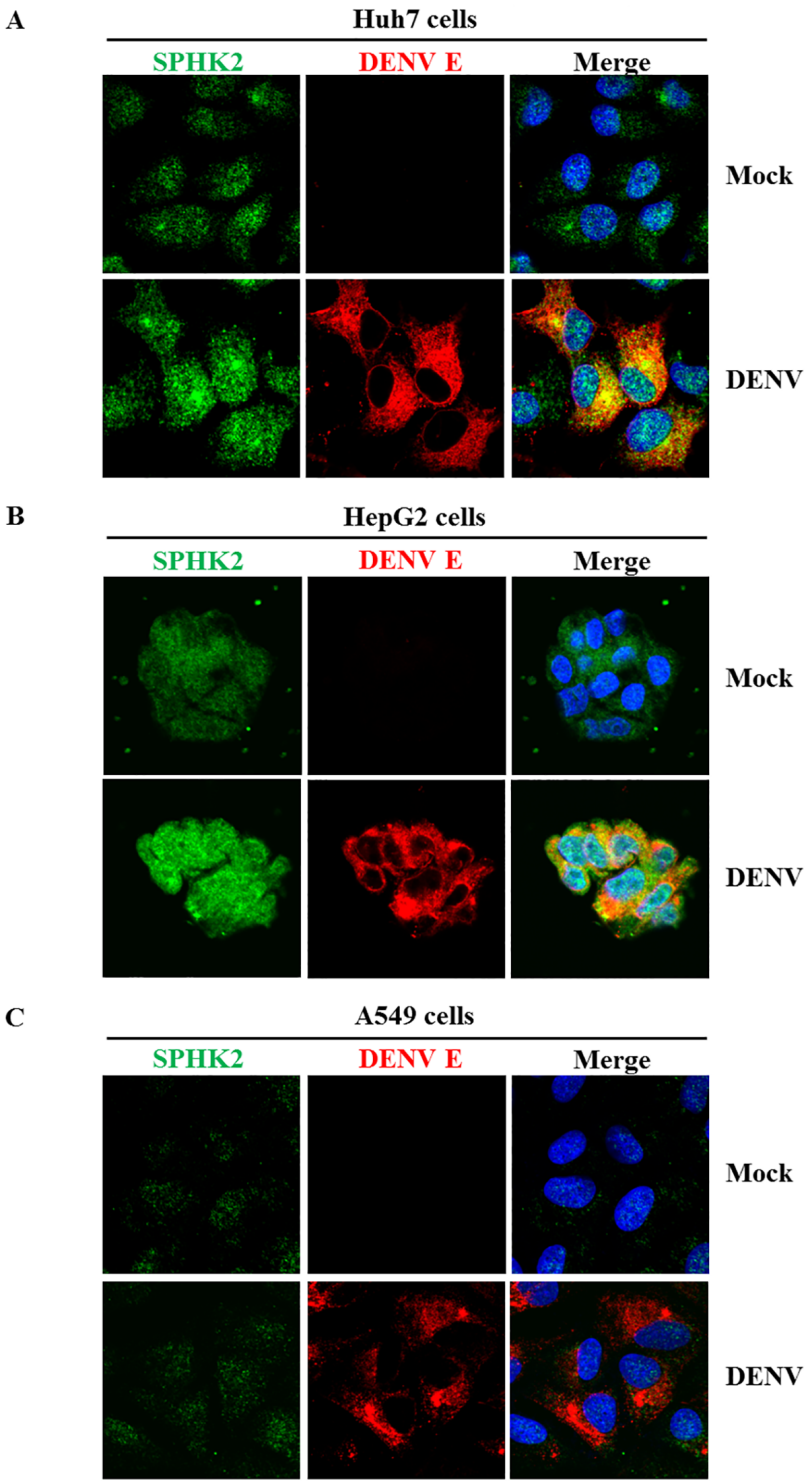
Alteration of SPHK2 protein expression and subcellular localization in DENV-infected Huh7 cells and HepG2 cells. Huh7 cells, HepG2 cells and A549 cells were infected with DENV at the MOI of 1, MOI of 5 and MOI of 1 for 24 hours, respectively. SPHK2 and DENV E proteins were detected by IFA and represented as green and red fluorescence, respectively. Hoechst 33342 was used to stain the nucleus. Mock cells (upper panel) and DENV-infected cells (lower panel) are (A) Huh7 cells (B) HepG2 cells and (C) A549 cells, respectively.

To determine whether the apoptosis role of *SPHK2* was restricted to the infected hepatic cell line, *SPHK2* knockdown and DENV infection experiments were additionally performed in HepG2 cells, another hepatic cell line and in A549, a lung cell line. Caspase 3 activity was elevated in both HepG2 cells and A549 cells upon infected with DENV suggesting the susceptibility of these cells in DENV-induced apoptosis (Fig 8A and 8B). Interestingly, knockdown of *SPHK2* significantly reduced the caspase 3 activity in infected HepG2 cells but failed to reduce that in infected-A549 cells (Fig 8A and 8B). These results suggest a role of *SPHK2* in DENV-induced apoptosis of liver cell lines. We next asked whether the expression and localization of SPHK2 in different cell lines may influence apoptosis during DENV infection. IFA double staining of SPHK2 and DENV E proteins was performed in Huh7 cells, HepG2 cells and A549 cells, respectively. Based on the fluorescent intensity, the basal level of SPHK2 protein expression was higher in Huh7 cells and HepG2 cells compared to that in A549 cells (Fig 9A, 9B and 9C). Up-regulation and altered subcellular localization of SPHK2 protein was also observed in DENV-infected liver cell lines compared to that in A549 cells (Fig 9A, 9B and 9C). Partial co-localization between DENV E and SPHK2 protein, especially in the perinuclear region, was evident in DENV-infected liver cell lines compared to that in A549 cells (Fig 9A, 9B and 9C). Data imply that expression and subcellular localization of SPHK2 may influence its role in apoptosis. The difference in expression and subcellular localization of SPHK2 between DENV-infected liver and lung cell lines may partly explain the different apoptotic phenotypes observed in this study. To conclude whether the apoptosis role of *SPHK2* was restricted to the infected liver cell lines, additional cell lines that also undergo apoptosis during DENV infection should be further tested.

### *SPHK2* knockdown in Huh7 cells infected with other serotypes of DENV show reduced apoptosis

**Fig 10:**
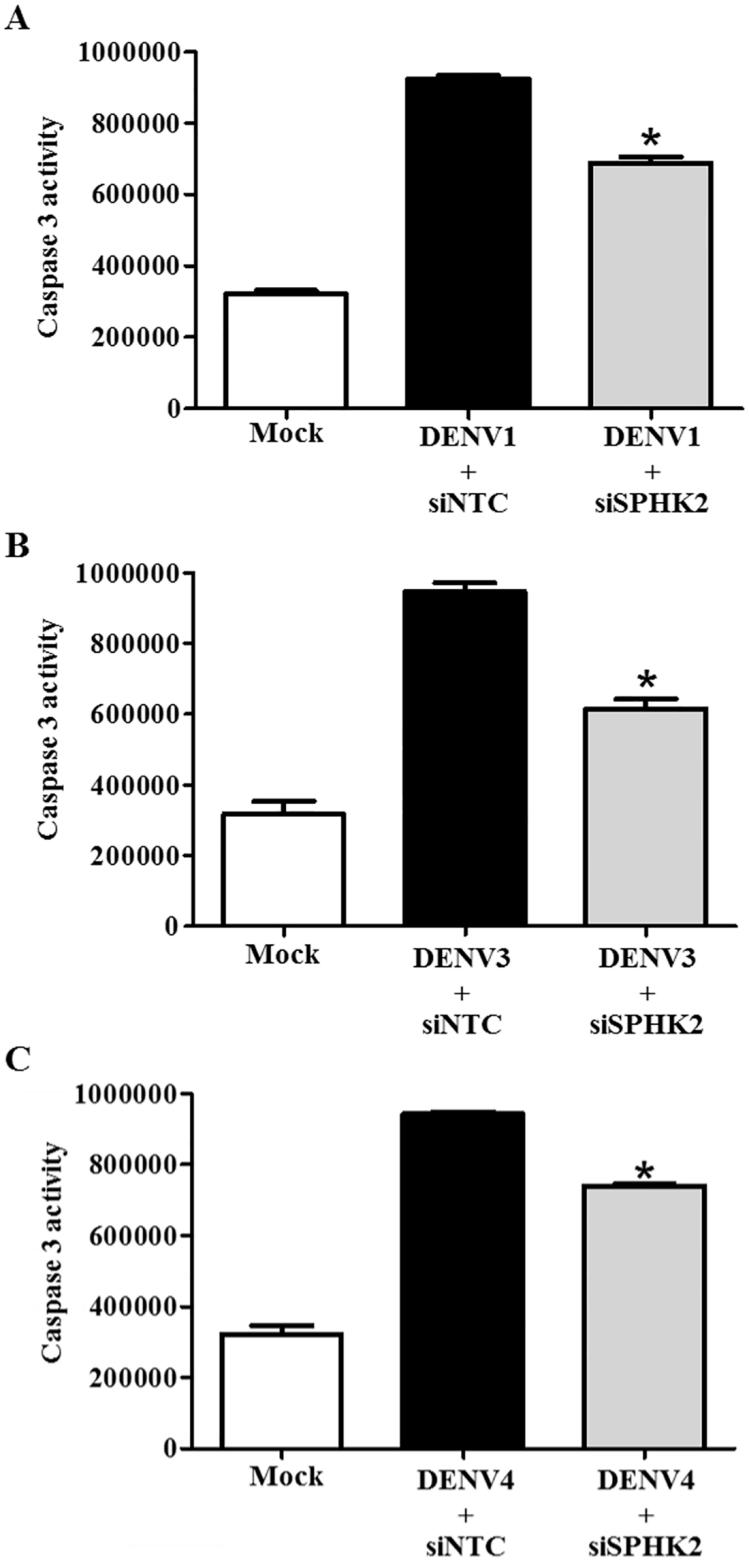
*SPHK2* knockdown reduces apoptosis in the other three serotypes of DENV. Huh7 cells were reverse transfected with si*SPHK2* or siNTC for 24 hours and then infected with different serotypes of DENV at MOI 10 for 48 hours. Caspase activity was measured and is represented individually for (A) DENV1-; (B) DENV3-; and, (C) DENV4-infected Huh7 cells. The results are represented in relative luminescent unit (RLU). The results are expressed as the average of three independent experiments ± SD. The asterisks indicate statistically significant differences between groups (p < 0.05) (Student’s *t* test).

To determine whether *SPHK2* is involved in the apoptosis of Huh7 cells infected with the other three serotypes of DENV (DENV1, DENV3, and DENV4), *SPHK2* knockdown experiments were conducted in the other three serotypes of DENV. Caspase 3 activity was found to be elevated in Huh7 cells infected with the other three serotypes of DENV (Fig 10A, 10B and 10C). Transfection with siSPHK2 in Huh7 cells separately infected with DENV1, DENV3, and DENV4 show a significant decrease in caspase 3 activity. Data suggest that all four serotypes of DENV share the function of host protein SPHK2 to induce apoptosis at least in part of caspase-3 activity induction.

## Discussion

DENV infection induces caspase 3 activity and apoptosis [22, 30]. Apoptosis-related gene expression profiling in DENV-infected HepG2 cells [31] and animal models [13, 14] was previously conducted. In this study, we set forth to screen for a list of apoptosis-related genes using siRNA library screening based on the alteration of the level of caspase 3 activity. The screening identified several genes, which involved in apoptosis of DENV-infected Huh7 cells. Knockdown of *TNFSF12* shows the strongest reduction of caspase 3 activity. *TNFSF12*, which is known as TNF-related weak inducer of apoptosis (*TWEAK*), induces apoptosis extrinsically by binding to its receptor on the cell surface [32]. *TNFSF12* was shown to be upregulated in DENV-infected HepG2 cells [33]. *TNFSF12* was also identified as the gene involved in influenza virus-induced apoptosis in lung cells [34]. However, transfection of siRNA targeting *TNFSF12* gene did not reduce DENV-induced apoptosis determined by Annexin V/PI staining (Fig 4A and 4B). The failure in reducing DENV-induced apoptosis with siRNA targeting *TNFSF12* gene could be related to the minimal presence of TNFSF12 protein after knocking down can still function to maintain apoptosis phenotype. Kinase enzymes are the majority of the identified genes in this study. Among those kinase genes, *RIPK2* was shown to be upregulated in DENV-infected HepG2 cells [33], and upregulated expression of *RIPK2* is previously shown to be essential for DENV-induced apoptosis [31]. *RIPK2* is capable of mediating apoptosis via several transduction pathways, including the NF-κB, p38, and JNK signaling pathways [35, 36]. In addition, *RIPK2* is directly activated via caspase 1 [37], which is reported to be essential for DENV-induced cell death [38]. In the present study, the apoptotic role of *RIPK2* in DENV-induced apoptosis was verified in siRNA library screening of DENV-infected Huh7 cells, suggesting its importance in DENV-mediated apoptosis of liver cells. As kinase enzymes are the majority of the identified genes in this study and *SPHK2* was shown to be upregulated in DENV-infected HepG2 cells [33], *SPHK2* was selected as the candidate kinase for functional analysis.

Sphingosine kinases (*SPHKs*) are sphingosine lipid metabolism enzymes, which specifically catalyze the phosphorylation of sphingosine to generate sphingosine-1-phosphate (S1P), a bioactive sphingolipid [39]. Up to date, two isoforms of *SPHKs* were characterized including *SPHK1* and *SPHK2* [40, 41]. S1P generated from *SPHK1* mediates pro-survival signals either by intracellularly activation of NF-kB [42] or extracellularly activation of the ERK1/2, PI3K/AKT, and PLC pathways [43, 44]. *SPHK1* can act as the anti-apoptotic molecule in DENV-infected HEK293 cells, where reduced *SPHK1* activity enhanced cell death [45, 46]. However, knock down of *SPHK1* in DENV-infected Huh7 cells in the present study did not affect the level of caspase 3 activity (S2 Fig). The discrepancy may be originated from the different cell types as well as the different experiment conditions used in each study. *SPHK1* was also shown to modulate DENV infection by alteration of innate responses that regulate susceptibility to DENV infection [47]. Increased plasma level of S1P was also observed in DENV-infected patients [48, 49]. Unlike *SPHK1*, roles *of SPHK2* in both anti-apoptosis and pro-apoptosis are previously reported. *SPHK2* can be one of the targets for cancer treatment as *SPHK2* functions as the pro-survival molecule in tumor growth and metastasis [50–53]. Inhibition of *SPHK2* attenuated tumor growth by induction of caspase 3-mediated cell apoptosis via enhancing the degradation of anti-apoptotic protein Mcl-1, and increasing the expression of a pro-apoptotic protein Noxa [54]. In contrast, *SPHK2* can function as the pro-apoptosis molecule. Silencing of *SPHK2* decreased TNF-α-induced apoptosis in HEK-293 cells and mouse embryonic fibroblasts [55, 56]. In addition, the mesangial cells isolated from *SPHK2*-deficient mice exhibited the resistance to staurosporine-induced apoptosis [57]. Furthermore, inhibition of *SPHK2* significantly reduced liver damage and improved the survival rate of mice suffering hepatic ischemic reperfusion. This protective effect was specifically due to the reverse of mitochondrial permeabilization event in the intrinsic pathway of apoptosis [58]. Similarly, our study also demonstrates that knockdown of *SPHK2* significantly reduced the level of caspase 3 activity in DENV-infected Huh7 cells and the reduction in caspase 3 activity was correlated with the increasing of viable cells in the *SPHK2* inhibitor-treated DENV-infected Huh7 cells at 48 and 72 hours post infection (S3 Fig). Although the role of *SPHK2* in DENV-induced apoptosis was validated by at least three assays including caspase 3 activity, subG1 staining, and Annexin V/PI staining, primary necrotic cells within Annexin V-positive/PI-negative staining phenotype should be carefully interpreted as not only apoptotic but also primary necrotic cells can have Annexin V-positive/PI-negative staining phenotype [59]. The necrotic cells (Annexin V-positive/PI-positive) was further counted and the similar level of necrotic cells was observed between siNTC- and si*SPHK2*-transfected DENV-infected Huh7 cells (S4 Fig). In this study, knockdown of *SPHK2* did not reduce caspase 8 activity; however, did reduce caspase 9 activity, suggesting its involvement of *SPHK2* in the intrinsic pathway of apoptosis. As *SPHK2* also plays roles in TNF-α-mediated extrinsic pathway of apoptosis in several apoptosis models [55, 56], the contribution of *SPHK2* in TNF-α signaling and in the extrinsic pathway of DENV-induced apoptosis needs further investigations.

Up-regulation of SPHK2, subcellular localization of SPHK2, and its co-localization with DENV proteins may influence apoptosis in DENV-infected cells. Several studies demonstrates the relationship between the pro-apoptotic function and its subcellular localization of SPHK2 [60]. S1P from ER-localized SPHK2 serves as the fuel for ceramide synthesis [41]. Ceramide can induce lysosomal membrane permeabilization (LMP) thereby affecting the cathepsin protease release [61, 62]. ER stress, which is a common feature of DENV-infected cells [63, 64], also up-regulates expression of *SPHK2* [65]. The up-regulated ER-localized SPHK2 may produce ceramide to induce LMP leading to releasing of cathepsin protease. Our group previously reported the significance of cathepsin B, which mediates DENV-induced apoptosis via the intrinsic pathway in HepG2 cells [30]. Secondly, S1P from mitochondria-localized SPHK2 can induce Bak-dependent mitochondrial membrane permeabilization (MMP) induction and cytochrome c releasing [55, 66]. DENV infection was reported to influence the mitochondrial fission process to favor its own replication [67], whether S1P from mitochondria-localized SPHK2 contributes to DENV-induced apoptosis should be further investigated. Finally, S1P from nuclear-localized SPHK2 can bind and inhibit the function of histone deacetylase (HDAC) [68], which influences infection and apoptosis in other viruses [69]. Interactions of DENV capsid (DENV C) and Daxx was implicated in DENV-induce apoptosis [19], whether S1P from nuclear-localized SPHK2 influence apoptosis in the nucleus of infected cells via DENV C, Daxx, and HDAC merits further investigation. However, *SPHK2* may play the indirect role in DENV-induced apoptosis as proteomic study of DENV-infected Huh7 cells recently reveals several altered proteins related to apoptosis following DENV infection [33] but these proteins does not directly interact with SPHK2.

## Conclusion

Based on the caspase 3 activity, siRNA library screening platform identifies *SPHK2* as one of the candidate genes involved in the apoptosis of DENV-infected hepatic cells. Functional studies demonstrate that knockdown of *SPHK2* reduces DENV-induced caspase 3 activity and caspase 9 activity in Huh7 cells suggesting its pro-apoptotic role via intrinsic pathway. In addition, *SPHK2* specifically contributes to apoptosis of DENV-infected liver cell lines. Knockdown of *SPHK2* in Huh7 cells infected with the four serotypes of DENV shows similar results, explaining the vital role of *SPHK2* in contribution to DENV-induced apoptosis.

## Supporting information

S1 Fig ABC294640 treatment does not induce toxicity in Huh7 cells at the tested concentrations. Huh7 cells were treated with doses of 2.5, 5, and 10 μM of ABC294640 or 0.01% v/v DMSO for 48 hours. Huh7 cells, which were cultured in media alone, were maintained as a parental control. Cell toxicity was determined using Presto-Blue dye assay and spectrophotometry analysis. Percentage of cell viability compared to that of parental control is shown from the average of three independent experiments. Statistical analysis was analyzed using Student’s *t* test.
(TIF)

S2 Fig Knockdown of *SPHK1* does not reduce caspase 3 activity in DENV-infected Huh7 cells. In the screening assay, Huh7 cells were reverse transfected with siRNA directed against the *SPHK1* gene for 24 hours before being infected with DENV at MOI of 10 for 48 hours. Caspase 3 activity was measured and represented as RLU. The results are expressed as the average of triplicate experiments ± SD. Statistical analysis was analyzed using Student’s *t* test.
(TIF)

S3 Fig Treatment of ABC294640 increases cellular viability of DENV-infected Huh7 cells at 48 and 72 hours post infection. Huh7 cells were pre-treated with 0.01% v/v DMSO or 10 μM concentrations of ABC294640 for 2 hours. The treated cells were infected with DENV at MOI 10 and were cultured in the presence of corresponding concentrations for 48, 72 and 96 hours. Cellular viability was determined using Presto-Blue dye assay and spectrophotometry analysis. Percentage of cell viability compared to that of mock cells-treated with DMSO control is shown from the average of three independent experiments. The asterisks indicate statistically significant differences between groups (p < 0.05) (Student’s *t* test).
(TIF)

S4 Fig Comparison of necrotic cells (Annexin V+/PI+) between siNTC- and si*SPHK2*-transfected cells. Huh7 cells were reverse transfected with siRNA directed against *SPHK2* genes for 24 hours before being infected with DENV for 48 hours. Necrotic and apoptotic cells were determined by Annexin V/PI staining and flow cytometry analysis. Bar graph represented the percentage of necrotic cells (Annexin V+/PI+), which was plotted and compared between those of siNTC- and of si*SPHK2*-transfected cells. The results are expressed as the average of three independent experiments ± SD. Statistical analysis was analyzed using Student’s *t* test.
(TIF)

S1 Table List of 558 human genes targeted by apoptosis siRNA library, and the alteration level of caspase 3 activity after siRNA library screening in DENV-infected Huh7 cells. To explore the involvement of the apoptotic genes in DENV-infected Huh7 cells, human apoptosis siRNA library (Dharmacon) screening was performed in DENV-infected Huh7 cells. The full list of the alteration of caspase 3 activity upon siRNA transfection was shown in the S1 Table. The results were analyzed as the percentage of caspase 3 activity compared to siNTC-transfected cells.
(PDF)
