# Supplementary figures and images for: RNAi screen reveals a role of SPHK2 in dengue virus–mediated apoptosis in hepatic cell lines
Source: PLoS One. 2017 Nov 16;12(11):e0188121. doi: 10.1371/journal.pone.0188121 (PMC5690425; doi:10.1371/journal.pone.0188121)

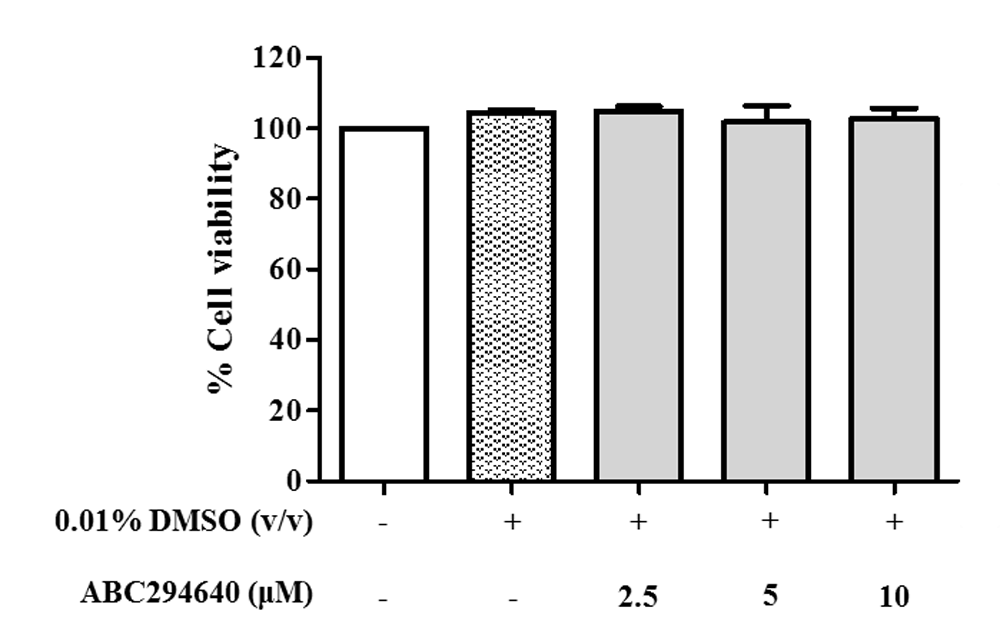

Supplement: S1 Fig — Huh7 cells were treated with doses of 2.5, 5, and 10 μM of ABC294640 or 0.01% v/v DMSO for 48 hours. Huh7 cells, which were cultured in media alone, were maintained as a parental control. Cell toxicity was determined using Presto-Blue dye assay and spectrophotometry analysis. Percentage of cell viability compared to that of parental control is shown from the average of three independent experiments. Statistical analysis was analyzed using Student’s t test. (TIF) [file pone.0188121.s001.tif]

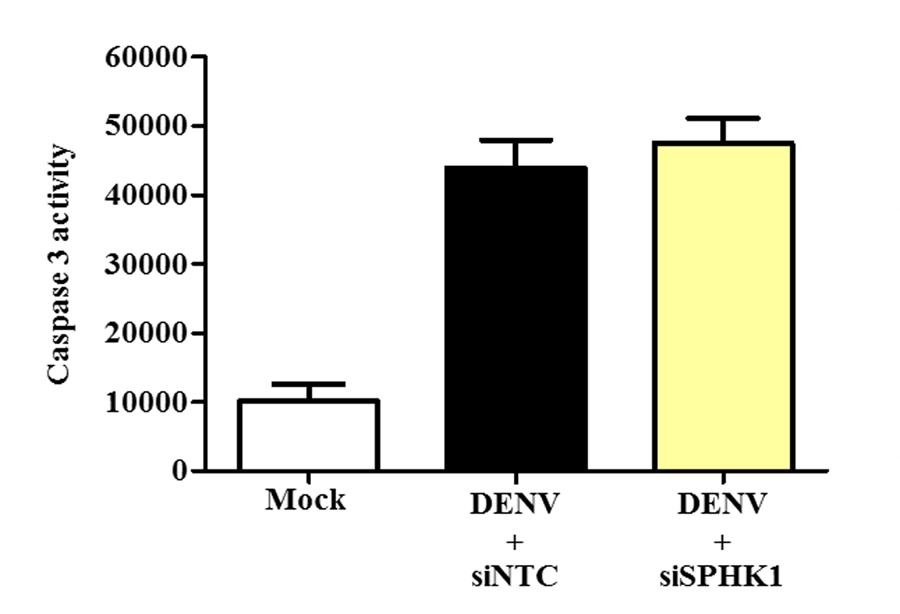

Supplement: S2 Fig — In the screening assay, Huh7 cells were reverse transfected with siRNA directed against the SPHK1 gene for 24 hours before being infected with DENV at MOI of 10 for 48 hours. Caspase 3 activity was measured and represented as RLU. The results are expressed as the average of triplicate experiments ± SD. Statistical analysis was analyzed using Student’s t test. (TIF) [file pone.0188121.s002.tif]

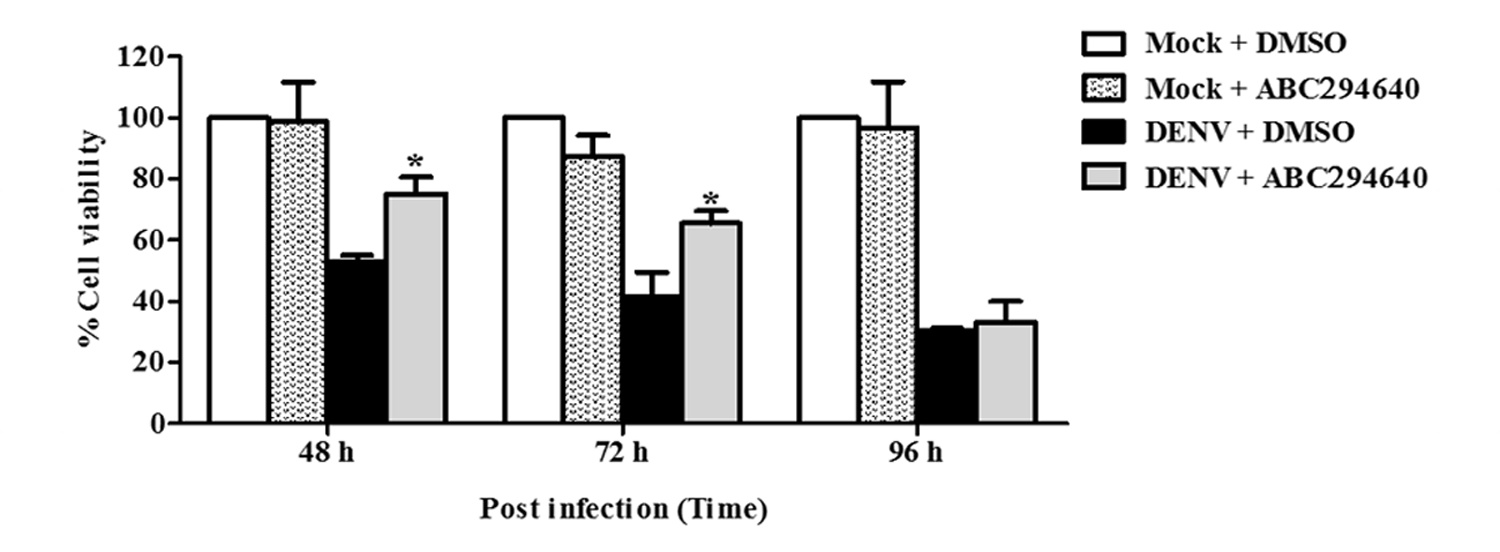

Supplement: S3 Fig — Huh7 cells were pre-treated with 0.01% v/v DMSO or 10 μM concentrations of ABC294640 for 2 hours. The treated cells were infected with DENV at MOI 10 and were cultured in the presence of corresponding concentrations for 48, 72 and 96 hours. Cellular viability was determined using Presto-Blue dye assay and spectrophotometry analysis. Percentage of cell viability compared to that of mock cells-treated with DMSO control is shown from the average of three independent experiments. The asterisks indicate statistically significant differences between groups (p < 0.05) (Student’s t test). (TIF) [file pone.0188121.s003.tif]

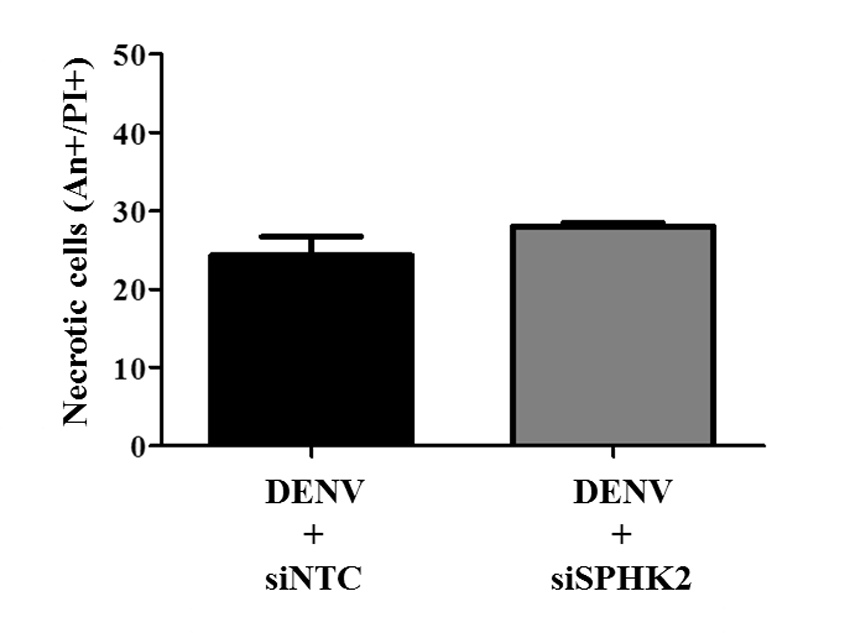

Supplement: S4 Fig — Huh7 cells were reverse transfected with siRNA directed against SPHK2 genes for 24 hours before being infected with DENV for 48 hours. Necrotic and apoptotic cells were determined by Annexin V/PI staining and flow cytometry analysis. Bar graph represented the percentage of necrotic cells (Annexin V+/PI+), which was plotted and compared between those of siNTC- and of siSPHK2-transfected cells. The results are expressed as the average of three independent experiments ± SD. Statistical analysis was analyzed using Student’s t test. (TIF) [file pone.0188121.s004.tif]
