# Supplementary material for: RNAi screen reveals a role of SPHK2 in dengue virus–mediated apoptosis in hepatic cell lines
Source: PLoS One. 2017 Nov 16;12(11):e0188121. doi: 10.1371/journal.pone.0188121 (PMC5690425; doi:10.1371/journal.pone.0188121)
Supplement: S1 Table — To explore the involvement of the apoptotic genes in DENV-infected Huh7 cells, human apoptosis siRNA library (Dharmacon) screening was performed in DENV-infected Huh7 cells. The full list of the alteration of caspase 3 activity upon siRNA transfection was shown in the S1 Table. The results were analyzed as the percentage of caspase 3 activity compared to siNTC-transfected cells. (PDF) [file pone.0188121.s005.pdf]

Dharmacon ON-TARGETplus® SMART pool® siRNA Library- Human Apoptosis
G-103900-E2 Lot 13121 (558 genes)

v	Well	Gene symbol	GENE ID	GI Number	Gene Accession No.	Caspase 3 activity (% to NTC)
		NTC				100.00
Plate 1	B03	ALB	213	8392890	NM_000477	69.89
Plate 1	B04	NOD1	10392	5174616	NM_006092	107.10
Plate 1	B05	ALOX12	239	4502050	NM_000697	109.68
Plate 1	B06	CARD6	84674	16554563	NM_032587	75.27
Plate 1	B07	ALOX15B	247	4557308	NM_001141	68.59
Plate 1	B08	CARD8	22900	7662403	NM_014959	80.13
Plate 1	B09	AIFM2	84883	31563505	NM_032797	101.62
Plate 1	B10	CARD9	64170	47717130	NM_052813	100.03
Plate 1	B11	ANGPTL4	51129	21536395	NM_016109	97.96
Plate 1	B12	CASP1	834	73622117	NM_033295	92.47
Plate 1	B13	ANXA1	301	4502100	NM_000700	91.08
Plate 1	B14	CASP10	843	47078268	NM_032974	94.28
Plate 1	B15	ANXA4	307	4809272	NM_001153	101.59
Plate 1	B16	CASP14	23581	6912285	NM_012114	83.55
Plate 1	B17	ANXA5	308	4809273	NM_001154	86.39
Plate 1	B18	CASP2	835	39995060	NM_032983	98.46
Plate 1	B19	APAF1	317	32483362	NM_181869	86.09
Plate 1	B20	CASP3	836	73622122	NM_032991	55.20
Plate 1	B21	ATG12	9140	38261968	NM_004707	81.16
Plate 1	B22	CASP4	837	73622123	NM_001225	84.92
Plate 1	C03	DDAH2	23564	7524353	NM_013974	95.27
Plate 1	C04	HSPA1B	3304	26787974	NM_005346	102.23
Plate 1	C05	DDX41	51428	21071031	NM_016222	90.62
Plate 1	C06	HSPA5	3309	21361242	NM_005347	54.49
Plate 1	C07	DEDD	9191	14670395	NM_004216	70.91
Plate 1	C08	HSPA9	3313	39812281	NM_004134	64.15
Plate 1	C09	DFFA	1676	47132578	NM_004401	58.32
Plate 1	C10	HSPB1	3315	4996892	NM_001540	69.03
Plate 1	C11	DFFB	1677	51988888	NM_004402	79.04
Plate 1	C12	HSPD1	3329	41399284	NM_199440	105.34

Plate 1	C13	DIABLO	56616	42544196	NM_138929	92.41
Plate 1	C14	HTATIP2	10553	34147574	NM_006410	92.97
Plate 1	C15	DNAJA3	9093	40786390	NM_005147	114.18
Plate 1	C16	IAPP	3375	4557654	NM_000415	71.13
Plate 1	C17	DNASE1	1773	58331227	NM_005223	96.50
Plate 1	C18	CARD18	59082	38683856	NM_021571	87.53
Plate 1	C19	DNASE1L3	1776	58331226	NM_004944	78.30
Plate 1	C20	IER3	8870	16554595	NM_003897	82.09
Plate 1	C21	DNASE2	1777	58331228	NM_001375	90.06
Plate 1	C22	IFI16	3428	5031778	NM_005531	97.86
Plate 1	D03	ATG5	9474	4757797	NM_004849	77.51
Plate 1	D04	CASP5	838	4757913	NM_004347	76.61
Plate 1	D05	API5	8539	41393590	NM_006595	87.87
Plate 1	D06	CASP6	839	73622127	NM_032992	66.31
Plate 1	D07	APLP1	333	67782339	NM_005166	63.06
Plate 1	D08	CASP7	840	73623014	NM_033340	53.77
Plate 1	D09	APOE	348	48762938	NM_000041	67.07
Plate 1	D10	CASP8	841	73623022	NM_033358	68.42
Plate 1	D11	APP	351	41406056	NM_201414	70.82
Plate 1	D12	CASP8AP2	9994	16306505	NM_012115	85.89
Plate 1	D13	NAE1	8883	66363687	NM_001018160	93.62
Plate 1	D14	CASP9	842	14790127	NM_032996	73.15
Plate 1	D15	APTX	54840	28329426	NM_175071	72.67
Plate 1	D16	CBX4	8535	55770829	NM_003655	92.94
Plate 1	D17	ARHGDIA	396	34147601	NM_004309	103.26
Plate 1	D18	CCL2	6347	56119169	NM_002982	84.77
Plate 1	D19	ARHGEF6	9459	47078220	NM_004840	98.45
Plate 1	D20	CD14	929	4557416	NM_000591	107.12
Plate 1	D21	PYCARD	29108	22035621	NM_145183	78.06
Plate 1	D22	CD2	914	156071471	NM_001767	76.36
Plate 1	E03	DNM2	1785	56549124	NM_001005362	39.94
Plate 1	E04	IFIH1	64135	27886567	NM_022168	61.11
Plate 1	E05	DOCK1	1793	50345995	NM_001380	67.58
Plate 1	E06	IFNA2	3440	11067750	NM_000605	86.68

Plate 1	E07	DPF1	8193	4758797	NM_004647	101.86
Plate 1	E08	IFNB1	3456	50593016	NM_002176	90.34
Plate 1	E09	DPF2	5977	21536317	NM_006268	91.08
Plate 1	E10	IGF1R	3480	11068002	NM_000875	85.45
Plate 1	E11	DUSP22	56940	34147625	NM_020185	47.90
Plate 1	E12	IGFBP3	3486	62243067	NM_000598	78.07
Plate 1	E13	DUSP6	1848	42764686	NM_022652	89.65
Plate 1	E14	IP6K2	51447	55769523	NM_001005911	48.26
Plate 1	E15	E2F1	1869	12669910	NM_005225	104.14
Plate 1	E16	IP6K3	117283	78191796	NM_054111	133.98
Plate 1	E17	EAF2	55840	41350199	NM_018456	81.31
Plate 1	E18	IKBKG	8517	21361093	NM_003639	104.33
Plate 1	E19	EBAG9	9166	37694064	NM_198120	55.27
Plate 1	E20	IL10	3586	24430216	NM_000572	64.00
Plate 1	E21	EDAR	10913	11641230	NM_022336	97.59
Plate 1	E22	IL17A	3605	27477085	NM_002190	93.14
Plate 1	F03	ATG3	64422	34147490	NM_022488	90.87
Plate 1	F04	CD28	940	5453610	NM_006139	90.56
Plate 1	F05	ATG7	10533	5453667	NM_006395	71.17
Plate 1	F06	CD38	952	38454325	NM_001775	69.15
Plate 1	F07	AVEN	57099	56699476	NM_020371	95.76
Plate 1	F08	CD3E	916	50726997	NM_000733	66.31
Plate 1	F09	AXIN1	8312	31083143	NM_181050	82.23
Plate 1	F10	CD3G	917	4557428	NM_000073	77.06
Plate 1	F11	CSRNP1	64651	17136074	NM_033027	101.65
Plate 1	F12	CD5L	922	5174410	NM_005894	76.16
Plate 1	F13	AZU1	566	28416954	NM_001700	66.61
Plate 1	F14	CD74	972	68448536	NM_001025158	75.87
Plate 1	F15	BAD	572	14670387	NM_032989	67.78
Plate 1	F16	CDK11B	984	16332359	NM_033487	113.45
Plate 1	F17	BAG1	573	72187559	NM_004323	71.00
Plate 1	F18	CDC2L2	985	16357489	NM_033534	88.91
Plate 1	F19	BAG2	9532	6715587	NM_004282	78.00
Plate 1	F20	CDK5	1020	38454327	NM_004935	104.48

Plate 1	F21	BAG3	9531	62530382	NM_004281	97.01
Plate 1	F22	CDK5R1	8851	34304373	NM_003885	103.36
Plate 1	G03	EEF1E1	9521	20127469	NM_004280	61.68
Plate 1	G04	IL18	3606	27502389	NM_001562	106.32
Plate 1	G05	EGLN3	112399	31742485	NM_022073	97.94
Plate 1	G06	IL19	29949	30795209	NM_153758	93.42
Plate 1	G07	EI24	9538	55956767	NM_001007277	92.81
Plate 1	G08	IL1A	3552	27894329	NM_000575	112.40
Plate 1	G09	EIF2AK2	5610	4506102	NM_002759	92.70
Plate 1	G10	IL1B	3553	27894305	NM_000576	105.82
Plate 1	G11	EIF4G2	1982	4503538	NM_001418	106.48
Plate 1	G12	IL2	3558	28178860	NM_000586	143.32
Plate 1	G13	ELMO1	9844	18765701	NM_130442	100.02
Plate 1	G14	IL24	11009	31317245	NM_181339	86.37
Plate 1	G15	ELMO2	63916	33469944	NM_022086	62.95
Plate 1	G16	IL2RA	3559	4557666	NM_000417	85.33
Plate 1	G17	ELMO3	79767	19718770	NM_024712	107.59
Plate 1	G18	IL2RB	3560	23238195	NM_000878	100.81
Plate 1	G19	EMP1	2012	4503558	NM_001423	71.97
Plate 1	G20	IL3	3562	28416914	NM_000588	98.24
Plate 1	G21	EMP2	2013	42716292	NM_001424	87.95
Plate 1	G22	IL4	3565	27477091	NM_172348	90.92
Plate 1	H03	BAG4	9530	14574569	NM_004874	79.76
Plate 1	H04	CDKN1A	1026	17978496	NM_000389	79.16
Plate 1	H05	BAG5	9529	62548853	NM_001015048	78.33
Plate 1	H06	CDKN2A	1029	47132605	NM_058195	136.29
Plate 1	H07	BAK1	578	33457353	NM_001188	95.35
Plate 1	H08	CEBPG	1054	34452718	NM_001806	67.20
Plate 1	H09	BCAP29	55973	56549094	NM_001008406	91.67
Plate 1	H10	GULP1	51454	56550114	NM_016315	75.62
Plate 1	H11	BARD1	580	4557348	NM_000465	79.20
Plate 1	H12	CFL1	1072	49472823	NM_005507	79.41
Plate 1	H13	BBC3	27113	24475588	NM_014417	61.16
Plate 1	H14	CFLAR	8837	21361768	NM_003879	65.30

Plate 1	H15	BCAP31	10134	49472837	NM_005745	170.87
Plate 1	H16	CGB7	94027	15451749	NM_033142	84.14
Plate 1	H17	BCAR1	9564	282398124	NM_014567	79.96
Plate 1	H18	CIAPIN1	57019	10092672	NM_020313	91.20
Plate 1	H19	BCL10	8915	20336470	NM_003921	113.40
Plate 1	H20	NLRP3	114548	34878689	NM_183395	103.70
Plate 1	H21	BCL2	596	72198345	NM_000657	169.61
Plate 1	H22	CIDEA	1149	38158008	NM_001279	121.59
Plate 1	I03	EMP3	2014	4503562	NM_001425	99.08
Plate 1	I04	IL6	3569	10834983	NM_000600	101.01
Plate 1	I05	ENDOG	2021	53759133	NM_004435	94.31
Plate 1	I06	IL7	3574	28610152	NM_000880	78.69
Plate 1	I07	EP300	2033	50345996	NM_001429	96.34
Plate 1	I08	CARD17	440068	55925611	NM_001007232	128.91
Plate 1	I09	ERCC2	2068	40068510	NM_000400	113.61
Plate 1	I10	ING4	51147	38201669	NM_016162	120.10
Plate 1	I11	ERCC3	2071	4557562	NM_000122	87.98
Plate 1	I12	INHA	3623	9257223	NM_002191	159.39
Plate 1	I13	ERN2	10595	51921282	NM_033266	129.08
Plate 1	I14	INHBA	3624	62953137	NM_002192	82.87
Plate 1	I15	ESPL1	9700	39652623	NM_012291	91.92
Plate 1	I16	INS	3630	4557670	NM_000207	99.96
Plate 1	I17	F2	2147	5922005	NM_000506	95.34
Plate 1	I18	ITGB2	3689	4557885	NM_000211	78.34
Plate 1	I19	F2R	2149	6031164	NM_001992	95.57
Plate 1	I20	ITGB3BP	23421	27597074	NM_014288	83.94
Plate 1	I21	FADD	8772	22219473	NM_003824	128.79
Plate 1	I22	ZNF346	23567	38570153	NM_012279	81.82
Plate 1	J03	BCL2A1	597	14574570	NM_004049	73.97
Plate 1	J04	CIDEB	27141	7656978	NM_014430	78.81
Plate 1	J05	BCL2L1	598	20336333	NM_001191	132.69
Plate 1	J06	CIDEC	63924	23943903	NM_022094	65.31
Plate 1	J07	BCL2L10	10017	20336328	NM_020396	78.10
Plate 1	J08	CLCF1	23529	50726992	NM_013246	140.33

Plate 1	J09	BCL2L12	83596	20336331	NM_052842	82.69
Plate 1	J10	CLU	1191	42716296	NM_001831	93.09
Plate 1	J11	BCL2L13	23786	45243500	NM_015367	74.55
Plate 1	J12	CLUL1	27098	40316925	NM_199167	116.01
Plate 1	J13	BCL2L2	599	14574571	NM_004050	80.92
Plate 1	J14	COL4A3	1285	14165449	NM_031366	84.60
Plate 1	J15	BCLAF1	9774	7661957	NM_014739	92.39
Plate 1	J16	CARD16	114769	62953112	NM_001017534	75.12
Plate 1	J17	BCL2L14	79370	21040331	NM_138724	97.60
Plate 1	J18	CRADD	8738	51988883	NM_003805	127.60
Plate 1	J19	BECN1	8678	19923741	NM_003766	133.18
Plate 1	J20	LUC7L3	51747	52426742	NM_006107	90.30
Plate 1	J21	BFAR	51283	7706090	NM_016561	116.28
Plate 1	J22	CSE1L	1434	29029560	NM_177436	97.20
Plate 1	K03	FAF1	11124	19528654	NM_131917	104.05
Plate 1	K04	JMY	133746	22748860	NM_152405	82.47
Plate 1	K05	FAIM	55179	50726985	NM_018147	102.13
Plate 1	K06	KCNIP3	30818	78190494	NM_001034914	123.38
Plate 1	K07	FAIM2	23017	34101289	NM_012306	63.71
Plate 1	K08	KNG1	3827	38455404	NM_000893	111.05
Plate 1	K09	FASTK	10922	39995106	NM_033015	95.59
Plate 1	K10	LALBA	3906	62739152	NM_002289	73.00
Plate 1	K11	FEM1B	10116	52851431	NM_015322	96.58
Plate 1	K12	LCK	3932	20428651	NM_005356	110.70
Plate 1	K13	FIS1	51024	151108472	NM_016068	83.46
Plate 1	K14	LGALS1	3956	6006015	NM_002305	113.10
Plate 1	K15	FKSG2	59347	11056001	NM_021631	72.47
Plate 1	K16	LGALS12	85329	20127658	NM_033101	110.64
Plate 1	K17	FASTKD1	79675	31377836	NM_024622	143.67
Plate 1	K18	LGALS7	3963	4504984	NM_002307	92.83
Plate 1	K19	FOSL2	2355	44680151	NM_005253	90.21
Plate 1	K20	LIMS1	3987	47078283	NM_004987	85.12
Plate 1	K21	FOXO1	2308	9257221	NM_002015	111.54
Plate 1	K22	LITAF	9516	65787264	NM_004862	58.47

Plate 1	L03	BID	637	37574727	NM_197967	102.96
Plate 1	L04	CTNNAL1	8727	4503128	NM_003798	57.13
Plate 1	L05	BIK	638	21536418	NM_001197	99.51
Plate 1	L06	CTNNBL1	56259	29570786	NM_030877	95.25
Plate 1	L07	NAIP	4671	4758751	NM_004536	118.46
Plate 1	L08	CTSB	1508	66346646	NM_001908	82.82
Plate 1	L09	BIRC2	329	41349435	NM_001166	74.87
Plate 1	L10	CUL1	8454	32307160	NM_003592	40.92
Plate 1	L11	BIRC3	330	33946284	NM_182962	86.21
Plate 1	L12	CUL2	8453	19482173	NM_003591	72.22
Plate 1	L13	XIAP	331	32528298	NM_001167	112.50
Plate 1	L14	CUL3	8452	45827792	NM_003590	78.30
Plate 1	L15	BIRC5	332	59859879	NM_001012270	52.67
Plate 1	L16	CUL4A	8451	57165422	NM_003589	96.17
Plate 1	L17	BIRC6	57448	61744455	NM_016252	108.66
Plate 1	L18	CUL5	8065	67514034	NM_003478	117.41
Plate 1	L19	BIRC7	79444	21536419	NM_022161	117.68
Plate 1	L20	CXCR4	7852	56790928	NM_003467	118.71
Plate 1	L21	BIRC8	112401	44680138	NM_033341	100.31
Plate 1	L22	CYCS	54205	34328939	NM_018947	55.76
Plate 1	M03	FOXO3	2309	42519915	NM_201559	75.13
Plate 1	M04	PIDD	55367	61742785	NM_145887	88.46
Plate 1	M05	FXR1	8087	61835163	NM_001013438	105.55
Plate 1	M06	LTA	4049	6806892	NM_000595	91.50
Plate 1	M07	GABARAPL1	23710	56676368	NM_031412	80.01
Plate 1	M08	LTBR	4055	4505038	NM_002342	90.88
Plate 1	M09	GADD45A	1647	9790904	NM_001924	89.97
Plate 1	M10	LY86	9450	55775471	NM_004271	118.10
Plate 1	M11	GADD45B	4616	299782594	NM_015675	104.25
Plate 1	M12	LYZ	4069	4557893	NM_000239	179.53
Plate 1	M13	GADD45G	10912	9790905	NM_006705	91.91
Plate 1	M14	MADD	8567	18860869	NM_130474	125.22
Plate 1	M15	GAS2	2620	29540560	NM_005256	83.47
Plate 1	M16	MAEA	10296	62953128	NM_005882	105.83

Plate 1	M17	GDNF	2668	40549412	NM_199234	155.88
Plate 1	M18	MAGEH1	28986	18105051	NM_014061	77.54
Plate 1	M19	TNFAIP8	25816	7657123	NM_014350	102.51
Plate 1	M20	MAGI3	260425	23097339	NM_152900	84.12
Plate 1	M21	GLO1	2739	5729841	NM_006708	91.88
Plate 1	M22	MAL	4118	12408664	NM_022440	126.85
Plate 1	N03	BMF	90427	51558703	NM_001003943	91.40
Plate 1	N04	DAD1	1603	4503252	NM_001344	70.93
Plate 1	N05	BNIP1	662	153946402	NM_013978	85.10
Plate 1	N06	DAP	1611	4758119	NM_004394	123.41
Plate 1	N07	BNIP2	663	4757855	NM_004330	133.65
Plate 1	N08	DAP3	7818	16905527	NM_004632	71.27
Plate 1	N09	BNIP3	664	7669480	NM_004052	85.23
Plate 1	N10	DAPK1	1612	4826683	NM_004938	103.28
Plate 1	N11	BNIP3L	665	47078259	NM_004331	101.29
Plate 1	N12	DAPK2	23604	71774012	NM_014326	85.29
Plate 1	N13	BNIPL	149428	19923712	NM_138279	88.77
Plate 1	N14	DAPK3	1613	4557510	NM_001348	107.16
Plate 1	N15	BOK	666	34335395	NM_032515	119.29
Plate 1	N16	DIDO1	11083	71044475	NM_080796	95.38
Plate 1	N17	BRAF	673	33188458	NM_004333	80.56
Plate 1	N18	DAXX	1616	53828721	NM_001350	64.87
Plate 1	N19	BRCA1	672	63252875	NM_007298	126.15
Plate 1	N20	DBC1	1620	7657008	NM_014618	95.70
Plate 1	N21	BRCA2	675	4502450	NM_000059	111.47
Plate 1	N22	DCC	1630	4885174	NM_005215	107.56
Plate 1	O03	GLRX2	51022	37537703	NM_197962	83.68
Plate 1	O04	MALT1	10892	27886565	NM_173844	70.55
Plate 1	O05	GML	2765	4504032	NM_002066	101.56
Plate 1	O06	MAP3K10	4294	21735549	NM_002446	113.92
Plate 1	O07	GPR65	8477	33695103	NM_003608	105.83
Plate 1	O08	MAP3K5	4217	21536459	NM_005923	104.41
Plate 1	O09	NDUFA13	51079	21361821	NM_015965	90.41
Plate 1	O10	MAPK1	5594	75709179	NM_138957	105.62

Plate 1	O11	GSTP1	2950	6552334	NM_000852	109.13
Plate 1	O12	MAPK8	5599	20986522	NM_139049	142.06
Plate 1	O13	GZMA	3001	6996012	NM_006144	118.41
Plate 1	O14	MAPK8IP2	23542	21237774	NM_139124	120.56
Plate 1	O15	GZMB	3002	32483414	NM_004131	132.99
Plate 1	O16	MARK4	57787	33636755	NM_031417	97.48
Plate 1	O17	GZMH	2999	31542869	NM_033423	117.29
Plate 1	O18	MCL1	4170	33519457	NM_182763	96.19
Plate 1	O19	GZMM	3004	7108347	NM_005317	133.39
Plate 1	O20	MDM4	4194	4505138	NM_002393	140.83
Plate 1	O21	HTT	3064	66934964	NM_002111	85.34
Plate 1	O22	PDCD2L	84306	14150140	NM_032346	152.61
Plate 2	B03	AATF	26574	76159274	NM_012138	83.67
Plate 2	B04	BTG1	694	4502472	NM_001731	60.35
Plate 2	B05	ABL1	25	62362413	NM_005157	55.92
Plate 2	B06	BTK	695	4557376	NM_000061	77.36
Plate 2	B07	ACIN1	22985	7662237	NM_014977	82.38
Plate 2	B08	CSRNP2	81566	13540601	NM_030809	93.10
Plate 2	B09	ACVR1C	130399	161333838	NM_145259	89.43
Plate 2	B10	TRIB3	57761	41327717	NM_021158	83.56
Plate 2	B11	ADORA1	134	4501946	NM_000674	103.01
Plate 2	B12	CALR	811	5921996	NM_004343	86.26
Plate 2	B13	ADORA2A	135	17136146	NM_000675	107.61
Plate 2	B14	CARD10	29775	51093860	NM_014550	78.07
Plate 2	B15	ADRA1A	148	15451760	NM_033304	76.14
Plate 2	B16	CARD11	84433	16507951	NM_032415	56.23
Plate 2	B17	AGTR2	186	23238239	NM_000686	68.93
Plate 2	B18	NLRC4	58484	40788014	NM_021209	39.05
Plate 2	B19	AHR	196	5016091	NM_001621	69.64
Plate 2	B20	CARD14	79092	16507954	NM_052819	48.12
Plate 2	B21	AKT1	207	62241012	NM_001014431	32.71
Plate 2	B22	NOD2	64127	11545911	NM_022162	88.83
Plate 2	C03	HDAC1	3065	13128859	NM_004964	83.00
Plate 2	C04	FASTKD3	79072	40068496	NM_024091	77.02

Plate 2	C05	HDAC3	8841	13128861	NM_003883	48.82
Plate 2	C06	MIF	4282	4505184	NM_002415	71.05
Plate 2	C07	HIP1	3092	38045918	NM_005338	73.05
Plate 2	C08	FOXO4	4303	5174578	NM_005938	92.63
Plate 2	C09	HIPK2	28996	46852175	NM_022740	82.93
Plate 2	C10	MMD	23531	52630444	NM_012329	85.72
Plate 2	C11	HIPK3	10114	29469068	NM_005734	83.86
Plate 2	C12	MMD2	221938	46048200	NM_198403	34.91
Plate 2	C13	HMGB1	3146	31982879	NM_002128	86.55
Plate 2	C14	MOAP1	64112	73747827	NM_022151	59.31
Plate 2	C15	HRK	8739	4504492	NM_003806	81.31
Plate 2	C16	MPO	4353	4557758	NM_000250	43.42
Plate 2	C17	FAM215A	23591	51474496	XM_499554	55.03
Plate 2	C18	MRPS30	10884	58331149	NM_016640	69.62
Plate 2	C19	C2orf28	51374	47078299	NM_080592	61.54
Plate 2	C20	MTL5	9633	40806199	NM_004923	81.01
Plate 2	C21	SEPT4	5414	17986246	NM_080416	84.90
Plate 2	C22	MTFP1	51537	51243056	NM_001003704	73.19
Plate 2	D03	MX1	4599	18490989	NM_002462	71.11
Plate 2	D04	NGFRAP1	27018	7657043	NM_014380	41.82
Plate 2	D05	MYBL2	4605	31652260	NM_002466	63.25
Plate 2	D06	NME1	4830	38045911	NM_000269	73.50
Plate 2	D07	NLRP1	22861	74271813	NM_001033053	74.42
Plate 2	D08	NME3	4832	37693992	NM_002513	54.96
Plate 2	D09	NLRP12	91662	15193291	NM_033297	85.93
Plate 2	D10	NME5	8382	37622352	NM_003551	56.02
Plate 2	D11	NLRP2	55655	8923472	NM_017852	84.50
Plate 2	D12	NME6	10201	38564323	NM_005793	59.64
Plate 2	D13	NCKAP1	10787	45505152	NM_013436	60.74
Plate 2	D14	NOL3	8996	34147652	NM_003946	60.55
Plate 2	D15	NCR1	9437	27545441	NM_004829	84.08
Plate 2	D16	NOTCH2	4853	24041034	NM_024408	69.80
Plate 2	D17	NFKB1	4790	34577121	NM_003998	93.85
Plate 2	D18	NPM1	4869	83641869	NM_001037738	45.41

Plate 2	D19	NFKBIA	4792	10092618	NM_020529	71.45
Plate 2	D20	NRG2	9542	7669535	NM_013985	98.01
Plate 2	D21	NGFR	4804	4505392	NM_002507	94.66
Plate 2	D22	NTF3	4908	45359869	NM_002527	70.01
Plate 2	E03	NTN1	9423	4758839	NM_004822	79.88
Plate 2	E04	PAWR	5074	55769532	NM_002583	94.42
Plate 2	E05	NUDT2	318	22219466	NM_147173	96.05
Plate 2	E06	PAX3	5077	31563349	NM_013942	79.59
Plate 2	E07	NUP62	23636	34335245	NM_012346	81.79
Plate 2	E08	PAX7	5081	7524358	NM_013945	86.25
Plate 2	E09	OPA1	4976	18860830	NM_130831	64.00
Plate 2	E10	PCBP4	57060	14670372	NM_033010	81.93
Plate 2	E11	P2RX1	5023	27894283	NM_002558	58.17
Plate 2	E12	PDCD1	5133	4826889	NM_005018	51.28
Plate 2	E13	TP53AIP1	63970	11545826	NM_022112	65.89
Plate 2	E14	PDCD10	11235	22538793	NM_145860	45.26
Plate 2	E15	NUPR1	26471	6912569	NM_012385	58.27
Plate 2	E16	PDCD11	22984	70980548	NM_014976	68.77
Plate 2	E17	PAK1	5058	42794768	NM_002576	66.25
Plate 2	E18	PDCD2	5134	21735591	NM_002598	48.51
Plate 2	E19	PARP1	142	11496989	NM_001618	54.14
Plate 2	E20	PDCD4	27250	34304340	NM_014456	71.17
Plate 2	E21	PARP4	143	11496990	NM_006437	80.05
Plate 2	E22	PDCD5	9141	21735599	NM_004708	87.53
Plate 2	F03	PDCD6	10016	22027539	NM_013232	61.52
Plate 2	F04	PHB	5245	6031190	NM_002634	68.20
Plate 2	F05	PDCD6IP	10015	48255927	NM_013374	76.32
Plate 2	F06	PHLDA1	22822	83977458	NM_007350	78.64
Plate 2	F07	PDCD7	10081	22027540	NM_005707	70.50
Plate 2	F08	PHLPP1	23239	37221174	NM_194449	60.65
Plate 2	F09	AIFM1	9131	22202630	NM_145813	67.45
Plate 2	F10	PIK3R2	5296	48976048	NM_005027	58.50
Plate 2	F11	PDCL3	79031	51944951	NM_024065	143.68
Plate 2	F12	PIM1	5292	31543400	NM_002648	68.26

Plate 2	F13	PDE1B	5153	24431942	NM_000924	135.73
Plate 2	F14	PLAGL1	5325	37622889	NM_002656	66.87
Plate 2	F15	PDIA2	64714	5803118	NM_006849	64.69
Plate 2	F16	PLG	5340	4505880	NM_000301	95.20
Plate 2	F17	PEA15	8682	21359835	NM_003768	101.39
Plate 2	F18	PML	5371	67089153	NM_033247	31.76
Plate 2	F19	PECR	55825	19923816	NM_018441	70.77
Plate 2	F20	POGK	57645	22027479	NM_017542	77.18
Plate 2	F21	PGLYRP1	8993	4827035	NM_005091	59.81
Plate 2	F22	PPARD	5467	29171749	NM_177435	64.60
Plate 2	G03	PPM1F	9647	29826333	NM_014634	66.39
Plate 2	G04	PRKCZ	5590	75709227	NM_001033582	76.93
Plate 2	G05	PPP1R13B	23368	18699719	NM_015316	97.66
Plate 2	G06	PRLR	5618	40254435	NM_000949	60.05
Plate 2	G07	PPP1R15A	23645	157674362	NM_014330	75.83
Plate 2	G08	PROC	5624	4506114	NM_000312	89.53
Plate 2	G09	PPP2CA	5515	57222566	NM_002715	73.34
Plate 2	G10	PRODH	5625	19924110	NM_016335	87.60
Plate 2	G11	PPP2R1A	5518	32455242	NM_014225	74.65
Plate 2	G12	PROK2	60675	24475653	NM_021935	76.53
Plate 2	G13	PPP2R1B	5519	32455243	NM_181699	61.90
Plate 2	G14	HTRA2	27429	73747818	NM_145074	75.41
Plate 2	G15	PRF1	5551	45935369	NM_005041	94.41
Plate 2	G16	PSEN1	5663	7549814	NM_007319	101.90
Plate 2	G17	PRKAA1	5562	47458812	NM_006251	111.92
Plate 2	G18	PSEN2	5664	7108359	NM_012486	48.99
Plate 2	G19	PRKCA	5578	47157319	NM_002737	72.57
Plate 2	G20	PTEN	5728	73765543	NM_000314	87.29
Plate 2	G21	PRKCE	5581	47157326	NM_005400	60.28
Plate 2	G22	PTGER3	5733	38505189	NM_198720	78.37
Plate 2	H03	PTH	5741	39995098	NM_000315	67.17
Plate 2	H04	RFFL	117584	62865648	NM_001017368	57.72
Plate 2	H05	PTK2B	2185	27886587	NM_173175	69.48
Plate 2	H06	RHOB	388	42716309	NM_004040	121.24

Plate 2	H07	PTPN6	5777	34328901	NM_080548	85.50
Plate 2	H08	RIPK1	8737	57242760	NM_003804	71.74
Plate 2	H09	ERC1	23085	38045893	NM_178038	62.81
Plate 2	H10	RIPK2	8767	40255024	NM_003821	45.02
Plate 2	H11	RAD21	5885	5453993	NM_006265	53.45
Plate 2	H12	RIPK3	11035	40254843	NM_006871	88.74
Plate 2	H13	RAF1	5894	52486392	NM_002880	71.88
Plate 2	H14	RNF130	55819	38176162	NM_018434	73.63
Plate 2	H15	PPP1R13L	10848	63003906	NM_006663	83.66
Plate 2	H16	RNF34	80196	37595536	NM_025126	91.92
Plate 2	H17	RASA1	5921	12545405	NM_022650	54.71
Plate 2	H18	RNF7	9616	34304332	NM_183237	32.94
Plate 2	H19	RB1	5925	4506434	NM_000321	82.31
Plate 2	H20	ROCK1	6093	4885582	NM_005406	104.22
Plate 2	H21	RELA	5970	46430498	NM_021975	82.63
Plate 2	H22	MST4	51765	15011879	NM_016542	69.70
Plate 2	I03	RRAGA	10670	57863286	NM_006570	64.73
Plate 2	I04	SERPINB9	5272	34147494	NM_004155	92.77
Plate 2	I05	RTN4	57142	47519538	NM_007008	63.20
Plate 2	I06	SETX	23064	37620158	NM_015046	78.88
Plate 2	I07	RYBP	23429	24432049	NM_012234	80.08
Plate 2	I08	SFRP5	6425	8400734	NM_003015	79.87
Plate 2	I09	S100B	6285	5454033	NM_006272	66.98
Plate 2	I10	SGK1	6446	25168262	NM_005627	88.77
Plate 2	I11	SCARB1	949	33620766	NM_005505	86.88
Plate 2	I12	SGPL1	8879	31982935	NM_003901	117.27
Plate 2	I13	SCIN	85477	14916472	NM_033128	93.19
Plate 2	I14	SH3GLB1	51100	21359904	NM_016009	108.73
Plate 2	I15	SHISA5	51246	21703709	NM_016479	105.49
Plate 2	I16	SIAH1	6477	63148617	NM_003031	61.58
Plate 2	I17	SEMA4D	10507	33942063	NM_006378	72.48
Plate 2	I18	SIAH2	6478	55925659	NM_005067	70.57
Plate 2	I19	SEMA6A	57556	47132510	NM_020796	59.32
Plate 2	I20	SIPA1	6494	24497626	NM_006747	141.10

Plate 2	I21	SERPINB2	5055	4505594	NM_002575	70.01
Plate 2	I22	SIRT1	23411	13775598	NM_012238	71.88
Plate 2	J03	SIVA1	10572	11277469	NM_021709	57.09
Plate 2	J04	SPHK2	56848	21361698	NM_020126	54.68
Plate 2	J05	SLAMF7	57823	19923571	NM_021181	65.91
Plate 2	J06	SPIN2A	54466	117606319	NM_019003	58.02
Plate 2	J07	SLC25A6	293	27764862	NM_001636	125.75
Plate 2	J08	SPP1	6696	38146097	NM_000582	90.03
Plate 2	J09	SMNDC1	10285	21361283	NM_005871	111.29
Plate 2	J10	SQSTM1	8878	46251280	NM_003900	68.86
Plate 2	J11	SNCA	6622	6806897	NM_007308	57.65
Plate 2	J12	SST	6750	71979669	NM_001048	52.20
Plate 2	J13	SOCS2	8835	21536304	NM_003877	89.61
Plate 2	J14	SSTR3	6753	44890055	NM_001051	91.45
Plate 2	J15	SOCS3	9021	45439351	NM_003955	100.15
Plate 2	J16	STAT1	6772	21536300	NM_139266	62.81
Plate 2	J17	SON	6651	21040319	NM_138925	164.54
Plate 2	J18	STK17A	9263	4758191	NM_004760	47.46
Plate 2	J19	SPATA4	132851	31543824	NM_144644	118.44
Plate 2	J20	STK17B	9262	217416412	NM_004226	57.00
Plate 2	J21	SPHK1	8877	21361087	NM_021972	108.14
Plate 2	J22	STK3	6788	5454093	NM_006281	107.47
Plate 2	K03	STK4	6789	38327560	NM_006282	68.97
Plate 2	K04	TGFB2	7042	4507462	NM_003238	78.89
Plate 2	K05	SULF1	23213	29789063	NM_015170	92.19
Plate 2	K06	THY1	7070	19923361	NM_006288	57.35
Plate 2	K07	CSRNP3	80034	23346411	NM_024969	98.67
Plate 2	K08	TIA1	7072	11863160	NM_022037	95.17
Plate 2	K09	TAOK2	9344	45505128	NM_004783	63.25
Plate 2	K10	TIAF1	9220	42741656	NM_004740	77.43
Plate 2	K11	TAX1BP1	8887	21361681	NM_006024	77.09
Plate 2	K12	TIAL1	7073	77695910	NM_003252	71.12
Plate 2	K13	TBRG4	9238	40217809	NM_199122	64.52
Plate 2	K14	TIMP3	7078	75905820	NM_000362	67.34

Plate 2	K15	TDGF1	6997	4507424	NM_003212	65.79
Plate 2	K16	TLR2	7097	68160956	NM_003264	104.91
Plate 2	K17	TMBIM6	7009	4507432	NM_003217	90.68
Plate 2	K18	TNF	7124	25952110	NM_000594	96.42
Plate 2	K19	TESK2	10420	6005895	NM_007170	65.76
Plate 2	K20	TNFAIP3	7128	26051241	NM_006290	73.64
Plate 2	K21	TGFB1	7040	63025221	NM_000660	103.70
Plate 2	K22	TNFRSF10A	8797	21361085	NM_003844	92.43
Plate 2	L03	TNFRSF10B	8795	22547118	NM_147187	70.15
Plate 2	L04	TNFRSF21	27242	23238206	NM_014452	69.67
Plate 2	L05	TNFRSF10C	8794	22547120	NM_003841	58.23
Plate 2	L06	CD40	958	23312370	NM_152854	75.98
Plate 2	L07	TNFRSF10D	8793	42544227	NM_003840	98.86
Plate 2	L08	FAS	355	23510430	NM_152876	92.16
Plate 2	L09	TNFRSF11B	4982	22547122	NM_002546	67.67
Plate 2	L10	TNFRSF6B	8771	14790166	NM_003823	43.47
Plate 2	L11	TNFRSF12A	51330	7706185	NM_016639	91.20
Plate 2	L12	CD27	939	23510435	NM_001242	67.00
Plate 2	L13	TNFRSF14	8764	23200040	NM_003820	126.23
Plate 2	L14	TNFRSF9	3604	23510438	NM_001561	85.39
Plate 2	L15	TNFRSF18	8784	23238193	NM_148901	85.11
Plate 2	L16	TNFSF10	8743	23510439	NM_003810	80.81
Plate 2	L17	TNFRSF19	55504	23238201	NM_018647	75.85
Plate 2	L18	TNFSF12	8742	23510442	NM_003809	44.25
Plate 2	L19	TNFRSF1A	7132	23312372	NM_001065	87.04
Plate 2	L20	TNFSF14	8740	25952146	NM_172014	77.16
Plate 2	L21	TNFRSF1B	7133	23312365	NM_001066	113.12
Plate 2	L22	TNFSF15	9966	23510444	NM_005118	85.13
Plate 2	M03	TNFSF18	8995	40354198	NM_005092	66.46
Plate 2	M04	TP73	7161	4885644	NM_005427	97.23
Plate 2	M05	CD40LG	959	58331233	NM_000074	80.58
Plate 2	M06	TP63	8626	31543817	NM_003722	151.73
Plate 2	M07	FASLG	356	4557328	NM_000639	87.09
Plate 2	M08	TPD52L1	7164	51173747	NM_001003397	92.67

Plate 2	M09	CD70	970	24119161	NM_001252	113.12
Plate 2	M10	TRADD	8717	24234723	NM_003789	101.43
Plate 2	M11	TNFSF8	944	24119162	NM_001244	63.99
Plate 2	M12	TRAF1	7185	53759116	NM_005658	88.37
Plate 2	M13	TNFSF9	8744	24119163	NM_003811	100.63
Plate 2	M14	TRAF2	7186	42544228	NM_021138	92.25
Plate 2	M15	FAIM3	9214	34147517	NM_005449	67.73
Plate 2	M16	TRAF3	7187	22027615	NM_003300	104.70
Plate 2	M17	TP53	7157	8400737	NM_000546	73.33
Plate 2	M18	TRAF4	9618	22027623	NM_145751	96.34
Plate 2	M19	TP53BP2	7159	72534661	NM_001031685	63.50
Plate 2	M20	TRAF5	7188	77404347	NM_001033910	54.79
Plate 2	M21	TP53INP1	94241	20127661	NM_033285	89.60
Plate 2	M22	TRAF6	7189	22027628	NM_004620	92.16
Plate 2	N03	TRAF7	84231	45594313	NM_206835	95.32
Plate 2	N04	UBE4B	10277	38327033	NM_006048	91.26
Plate 2	N05	TRAIP	10293	40807468	NM_005879	90.29
Plate 2	N06	UNC13B	10497	38176153	NM_006377	92.01
Plate 2	N07	RNF216	54476	46370053	NM_019011	83.45
Plate 2	N08	UNC5A	90249	62243566	NM_133369	68.59
Plate 2	N09	TRIM35	23087	70608107	NM_015066	80.01
Plate 2	N10	UNC5B	219699	32261317	NM_170744	83.05
Plate 2	N11	SPATA3	130560	31712019	NM_139073	125.82
Plate 2	N12	UNC5C	8633	16933524	NM_003728	97.59
Plate 2	N13	TUBB	203068	34222261	NM_178014	109.33
Plate 2	N14	UNC5D	137970	18254471	NM_080872	79.38
Plate 2	N15	TUBB4B	10383	68051719	NM_006088	108.21
Plate 2	N16	UTP11L	51118	52856412	NM_016037	67.94
Plate 2	N17	TMX1	81542	34222132	NM_030755	109.85
Plate 2	N18	VCP	7415	7669552	NM_007126	87.64
Plate 2	N19	TXNDC5	81567	42794770	NM_030810	73.92
Plate 2	N20	VDAC1	7416	4507878	NM_003374	114.54
Plate 2	N21	TXNL1	9352	4759273	NM_004786	72.79
Plate 2	N22	VEGFA	7422	71051584	NM_001025370	86.39

Plate 2	O03	VHL	7428	38045905	NM_198156	70.21
Plate 2	O04	YARS	8565	38202242	NM_003680	89.15
Plate 2	O05	YWHAG	7532	21464100	NM_012479	80.82
Plate 2	O06	YWHAH	7533	61744461	NM_003405	65.29
Plate 2	O07	ZAK	51776	82880647	NM_016653	78.18
Plate 2	O08	ZBTB16	7704	66932931	NM_001018011	131.91
Plate 2	O09	ZDHHC16	84287	37594446	NM_198045	89.74
Plate 2	O10	ZNF443	10224	31543989	NM_005815	109.43
Plate 2	O11	DEDD2	162989	31377621	NM_133328	51.62
Plate 2	O12	HSP90B1	7184	4507676	NM_003299	117.31
Plate 2	O13	PTRH2	51651	62865877	NM_016077	103.56
Plate 2	O14	BAX	581	34335121	NM_138763	65.53
Plate 2	O15	BCL2L11	10018	46276878	NM_138627	85.87
Plate 2	O16	ERN1	2081	50346000	NM_001433	104.53
Plate 2	O17	MZB1	51237	117938313	NM_016459	119.99
Plate 2	O18	PRDX2	7001	33188451	NM_181737	91.34
Plate 2	O19	PTPRC	5788	18641361	NM_080921	91.71
Plate 2	O20	TNFRSF25	8718	23200030	NM_148970	97.34
